# Engineering of the High-Precision Cas12m Dual Base Editor-Loaded Virus-like Particles for A-to-G and C-to-T Conversions

**DOI:** 10.3390/genes17101247

**Published:** 2026-10-09

**Authors:** Timur Aliev, Almaz Imatdinov, Elena Prudnikova, Ilnaz Imatdinov

**Affiliations:** 1 State Research Center of Virology and Biotechnology “Vector”, Kol’tsovo 630559, Novosibirsk Region, Russia; imatdinov_ar@vector.nsc.ru (A.I.); prudnikova_eyu@vector.nsc.ru (E.P.); imatdinov_ir@vector.nsc.ru (I.I.); 2 Faculty of Natural Sciences, Novosibirsk State University, Novosibirsk 630090, Russia

**Keywords:** base editing, CRISPR/Cas, linker, Cas12m, Cas9, TadDE, VLP

## Abstract

**Background/Objectives**: CRISPR/Cas-dependent base editing enables the precise correction of genomic sequences by direct modification of DNA bases without creating potentially mutagenic DNA double-strand breaks. Base editing is considered a therapeutic approach for rare monogenic disorders or viral infections. However, many variants of base editors suffer from limited specificity because they induce bystander edits within a broad editing window. However, nucleotide substitution is sufficient to correct a mutation or to achieve a therapeutic effect. **Methods**: PCR, cloning by restriction digest, and ligation were used to create genetic constructs. The editing efficiency was evaluated using NGS. Virus-like particles were produced in HEK293T cells using transient expression. VLPs were visualized by electron microscopy. **Results**: Using dual base editors that induce C-to-T and A-to-G substitutions, we show that high editing precision can be achieved by engineering the linker between the MmCas12m domain and the deaminase domain of the editor. By testing different linker sequences, we have created high-precision dual base editors with narrowed editing windows that can edit a single nucleotide at a target position with high accuracy. **Conclusions**: We found that shortening the linker to an acceptable length provides a single-nucleotide substitution in vitro with minimal bystander activity (less than 0.8%), while the high-precision MmCas12m dual base editor retains up to 90% of the editing efficiency of the original base editor. These high-precision base editors will be useful in cases where high precision is required, and editing of adjacent nucleotides is undesirable.

## 1. Introduction

Editing DNA sequences within the genome of living cells makes it possible to introduce new features or correct mutations. RNA-programmable CRISPR-associated nucleases (Cas) have made a significant contribution to gene editing through the ability to introduce a double-stranded DNA break (DSB) at a genome location of interest [1,2,3,4,5]. However, DSB does not directly lead to DNA editing. Post-nuclease editing occurs because of cellular reactions to DSBs. In such cases, editing is carried out because of processes including non-homologous end-joining (NHEJ) and microhomology-mediated end-joining (MMEJ) [6,7]. During DNA repair, genomic rearrangements (insertions, deletions, or translocations) occur at the site of a DSB [8]. However, it is possible to introduce a donor DNA template encoding the necessary DNA change flanked by sequences homologous to the region upstream and downstream of the DSB. In this case, DNA repair follows the homology-directed repair (HDR) pathway, and the exogenous DNA template is incorporated at the DSB site. However, HDR is possible only in the G2 and S phases. In addition, NHEJ is more efficient than HDR under most conditions. Therefore, edited products usually contain insertions or deletions [9,10].

Despite the variety of mutations, the most numerous class of known human pathogenic mutations is the single-nucleotide polymorphism (SNP) [11]. In this regard, the correction of SNPs is of greater interest from the point of view of gene therapy. Cas-mediated DSBs result in indels and translocations that are undesirable when trying to correct a point mutation. Base editors are a promising genome editing tool that directly corrects point mutations in genomic DNA without DSBs or a DNA donor template, or cellular HDR [12]. DNA base editors (BEs) are composed of a catalytically impaired Cas nuclease and a deaminase that catalyze the hydrolytic deamination of bases in nucleotides [13]. Upon binding to the target locus, the editor induces the formation of an R-loop in the DNA. The deaminase enzyme modifies DNA bases within single-stranded DNA. To improve efficiency in eukaryotic cells, a nickase form of Cas nucleases is used. A nick in the non-edited DNA strand promotes the cell to repair the non-edited strand using the edited strand as a template [14,15]. There are various DNA BEs: cytosine base editors (CBEs) convert a C-G pair into a T-A pair, adenine base editors (ABEs) convert an A-T pair to a G-C pair, and dual base editors provide both types of substitutions [16].

The specificity of the base editors is low in terms of site selectivity. CBEs can potentially edit all C located in an approximately 4–9 nucleotide editing window within the protospacer [17,18,19]. ABEs have a similar editing window [20]. Mutagenesis of all nucleotides in the editing window can be justified if multiple mutagenesis is necessary. However, if the task is to introduce a single-nucleotide substitution, then it becomes necessary to engineer special high-precision variants of base editors. Introduction of mutations that reduce the deaminase activity led to a decrease in the width of the editing window [21,22,23]. At the same time, a decrease in activity is directly associated with a decrease in the therapeutic effect. A compromise in such cases may be to optimize the length of the linker and the location of the base editor domains relative to each other [19]. This strategy does not lead to sufficient inhibition of the deaminase activity, but only reduces the editing window. Inspired by this idea, we have created the high-precision MmCas12m-TadDE dual base editor. The MmCas12m protein, derived from *Mycolicibacterium mucogenicum*, presents several advantageous features for genetic engineering compared to the widely used Cas9 nucleases. Notably, MmCas12m recognizes a T-rich TTN protospacer-adjacent motif (PAM), thereby expanding the targetable genomic space beyond the reach of SpCas9. Unlike typical Cas9 nucleases, wild-type MmCas12m is inherently catalytically inactive. It possesses a compact architecture (596 amino acids), which facilitates efficient delivery via a single adeno-associated virus (AAV) vector and enhances packaging efficiency in VLPs. Furthermore, MmCas12m exhibits a higher binding affinity for target DNA than other Cas9 proteins. This strong binding is attributed to its native biological function, which involves repressing the transcription of exogenous nucleic acids through tight DNA binding rather than inducing double-strand breaks [24,25]. Additionally, we have fused MmCas12m with the new recombinant dual deaminase based on TadA-8e (TadDE). TadDE has the ability to deaminate adenine and cytosine according to the original study [26]. Here, we present the results of the study in which we obtained the high-precision MmCas12m dual base editor capable of replacing only one nucleotide in the editing window with minimal bystander effects.

## 2. Materials and Methods

### 2.1. Cells

HEK293T (Human embryonic kidney 293T, Cat. no. 273) cells were sourced from the cell culture collection of the State Research Center of Virology and Biotechnology "Vector" (Federal Budgetary Institution of Science, Russia). The cells were maintained in Dulbecco’s modified Eagle’s medium/F12 (DMEM/F12; BioloT, Moscow, Russia) supplemented with 10% heat-inactivated fetal bovine serum (FBS) and a penicillin–streptomycin solution (BioloT). Culturing was performed at 37 °C in a humidified incubator with 5% CO_2_.

### 2.2. Plasmids

The following plasmids were used for the study (Table 1).

Restriction endonucleases and DNA ligases used for cloning are from NEB (New England Biolabs, Orlando, FL, USA). All oligonucleotides were synthesized by Evrogen (Evrogen, Moscow, Russia). Nucleotide sequences of plasmids (Table 1) were confirmed by Sanger sequencing using a BigDye Terminator v3.1 Cycle Sequencing kit (Thermo Fisher Scientific, Waltham, MA, USA) on an ABI 3500/3500xl Genetic Analyzer (Applied Biosystems, Carlsbad, CA, USA). Plasmid DNA was purified using a Plasmid Miniprep kit (#T1010L, New England Biolabs). DNA fragments from the agarose gel were purified using a Cleanup Mini (#BC023L, Evrogen). The complete nucleotide sequences of the expression cassettes for all variants of base editors are shown in the Supplementary Materials Section.

pCas12-dBE, pdBE-Cas12, pCas9-dBE, and pdBE-Cas9 plasmids were based on the transfer plasmid pLenti CMV GFP Puro (#17448, Addgene, Watertown, MA, USA). The GFP reporter and regulatory sequences of the lentiviral system were removed from the original plasmid. Instead, the target nucleotide sequences of each base editor (Supplementary Figures S1–S4) were cloned under the control of the CMV promoter via PciI and AvrII sites. The TadDE and MmCas12m genes were synthesized de novo in our previous research. The sequence of the catalytically inactive SpCas9 (dSpCas9) gene was obtained from a donor plasmid (#201953, Addgene). Additionally, the specific U6-driven tracrRNA sequence (Supplementary Figure S5) of MmCas12m or SpCas9 was cloned into pCas12-dBE, pdBE-Cas12 or pCas9-dBE, and pdBE-Cas9, respectively. Two BsaI sites are located near the tracrRNA sequence for cloning any crRNA sequence. During transcription, synthesized sgRNA molecules consist of tracrRNA and crRNA parts. sgRNA oligonucleotides were ligated into pCas12-dBE, pdBE-Cas12, pCas9-dBE, and pdBE-Cas9 via BsaI sites. Variations in Cas12-dBE with different linkers (Supplementary Figure S6) were obtained based on pCas12-dBE.

pGag-MmCas12m-TadDE encodes an engineered HIV-1 Gag-Cas-deaminase-6×His fusion protein under the control of the CMV promoter. The nucleotide sequence coding for the fusion protein is shown in Supplementary Figure S7. pLentiPack was used as a basis for pGag-Cas12m-TadDE. The nucleotide sequence of the fusion proteins was cloned into pLentiPack via SphI and XcmI sites instead of the original sequence. psgRNA-Cas12m encodes a specific single guide RNA under the control of the U6 promoter. psgRNA-Cas12m was obtained based on a donor plasmid (#122089, Addgene) by replacing the original sequence with the target sequence (Supplementary Figure S5A) via PciI and KpnI sites.

### 2.3. Plasmid Transfection

Cells (HEK293T) were seeded in 12-well plates at a density of 180,000 per well in 1 mL of complete medium (DMEM/F12, 10% FBS). After 12 h, cells were transfected with 1000 ng of plasmid DNA and the transfection reagent Lipofectamine 2000 (Thermo Fisher Scientific) in FBS-free Opti-MEM medium according to the manufacturer’s protocol. Briefly, nucleic acid was diluted in Opti-MEM medium (50 μL) without serum or antibiotics. The transfection reagent Lipofectamine 2000 (3 μL) was diluted in Opti-MEM (50 μL) and incubated for 5 min at room temperature. Diluted nucleic acid and diluted Lipofectamine 2000 were combined and incubated for 20 min at room temperature. Formed complexes were added to wells containing cells in growth medium. After 8 h, the medium was replaced with fresh full medium. After 48 h, genomic DNA was isolated using the QIAamp DNA Investigator Kit (QIAGEN, Hilden, Germany).

### 2.4. Virus-like Particles Production

The production protocol described in the referenced research is used as the basis [15]. HEK293T cells were seeded in 10 cm dishes (TPP, Trasadingen, Switzerland) at a density of 3.2 × 10^6^ per plate in 10 mL of complete medium. The next day, cells were transfected with Lipofectamine 2000 (Thermo Fisher Scientific) according to the manufacturer’s protocol. For production of VLPs with MmCas12m-TadDE, cells were transfected with a mixture of plasmids pCMV-VSV-G (400 ng, #8454 Addgene), pLentiPack (3375 ng), pGag-MmCas12m-TadDE (1125 ng), and psgRNA-MmCas12m (4400 ng) in FBS-free Opti-MEM medium (Thermo Fisher Scientific). After 8 h, the medium was replaced with fresh production medium supplemented with 2% FBS (BioloT). The concentration was carried out according to the method described in the following reference [23]. Briefly, supernatants with VLPs (10 mL) were collected 48–50 h after transfection, centrifuged at 300× *g* for 3 min, filtered through a 0.45 μm PVDF filter (Millipore, Burlington, MA, USA), aliquoted into 2 mL tubes, and centrifuged at 21,000× *g* for 2.5 h (4 °C). A microcentrifuge with a fixed-angle rotor was used. Pellets with VLP samples were resuspended in Opti-MEM (Thermo Fisher Scientific), combined into one tube, and adjusted to 200 μL with Opti-MEM (approximate concentration by 50-fold). An aliquot (20 μL) of each sample was taken for Western blot, and the rest of the sample was treated with 1.8 μL of DNAse I (SibEnzyme, Berdsk, Russia) with the addition of the corresponding 10× DNAse I buffer by incubation at 37 °C for 10 min. VLP samples were stored at −70 °C.

For producing improved VLPs with MmCas12m-TadDE, a mixture of plasmids, including pCMV-VSV-G (400 ng, #8454 Addgene), pLentiPack (2925 ng), pGag-MmCas12m-TadDE (1575 ng), and psgRNA-MmCas12m (4400 ng), was co-transfected. For production of the “Kombi” VLP series, the equimolar ratio of plasmids psgRNA-MmCas12m was used. For example, 2200 + 2200 ng for Kombi-2125, 1450 + 1450 + 1450 ng for Kombi-21251, and 1100 + 1100 + 1100 + 1100 ng for Kombi-21251-SL.

### 2.5. VLP Electron Microscopy

A total of 10 μL of the sample was added to 200 mesh copper grids coated with a continuous carbon film. The sample was allowed to adsorb for 10 s. Excess solution was removed. Then, the grids were placed on a drop of uranyl acetate solution and incubated for 20 s. The grids were dried and analyzed. VLPs were visualized using a JEM-1400 electron microscope (JEOL Ltd., Tokyo, Japan) at an accelerating voltage of 80 kV.

### 2.6. Western Blot

To check the content of the MmCas12m dual base editor in VLPs, concentrated samples were lysed in a 4× SDS-PAGE buffer (250 mM Tris-HCl, pH 6.8, 40% glycerol, 8% SDS, 4% 2-mercaptoethanol, and 0.2% Bromphenol Blue) and incubated at 90 °C for 3 min.

SDS-PAGE was performed using 12% polyacrylamide gels in a Laemmli buffer system, followed by transfer onto a 0.45 µm nitrocellulose membrane (Bio-Rad, Hercules, CA, USA) using a power blotter system (#PB0012, Thermo Fisher Scientific). The 6×His-tagged MmCas12m-TadDE was detected using an anti-6×His tag mouse monoclonal antibody (#J099B12, BioLegend, San Diego, CA, USA); p24 HIV-1 (a loading control for VLP samples) was detected using an anti-p24 mouse antibody (#MA5-44993, Thermo Fisher Scientific). A horseradish peroxidase-conjugated goat anti-mouse antibody (#1706516, Bio-Rad) was used as a secondary antibody. The image was obtained with the Gel Doc XR+ Gel Documentation System (Bio-Rad).

### 2.7. VLP Treatment

A total of 5 × 10^4^ HEK293T cells in 50 µL were added to a dose of VLP, mixed by gentle pipetting, and cultured in a 96-well plate. After 8 h, the medium was replaced with fresh complete medium. Cells were cultured. After 48 h, genomic DNA was isolated using the QIAamp DNA Investigator Kit (QIAGEN).

### 2.8. NGS

All primers used for the amplification of the edited loci of the genome are presented in Supplementary Figure S8. The target loci were amplified using Phusion U Green Multiplex PCR Master Mix (Thermo Fisher Scientific) under the following conditions: 95 °C (3 min); 25 cycles of 95 °C (10 s), 58 °C (20 s) and 72 °C (20 s); and 72 °C (2 min). The PCR products were purified using a QIAquick Gel Extraction Kit (QIAGEN). The NGS library preparation was carried out using the NEBNext Ultra II FS DNA Library Prep Kit for Illumina (New England Biolabs). The sequencing was performed on the Illumina MiSeq platform (Illumina, San Diego, CA, USA).

The sequencing depth for each sample was at least 1000×. Primary processing of FASTQ files was performed using fastp v0.20.1 to remove adapter sequences and to filter reads by quality (Q score > 20). Reads were aligned to the reference AAVS1 locus (GenBank ID: AC010327.8) using BWA-MEM v. 0.7.18. Processing and analysis of aligned data in SAM/BAM formats were performed using Samtools v1.11. Consensus sequences were extracted from BAM files using iVar v1.2.2.

### 2.9. Statistical Analysis

One-way ANOVA followed by Tukey’s post hoc test was used to compare differences between groups (*n* = 3 in each group). The level of statistical significance was set at *p* < 0.05. Statistical analyses were conducted using GraphPad Prism 9 software (v 9.3.1, San Diego, CA, USA).

### 2.10. DNA Plasmid Detection in Genomic DNA Samples

To identify the residual plasmid DNA used for VLP production in the genomic DNA samples, we performed real-time PCR screening using specific primers. Pairs of primers were chosen for the VSV-G gene (F: 5′-ATGAAGTGCCTTTTGTACTTAAC-3′, R: 5′-CTGTGCCTATTAAGTCATTATGCCA-3′), the MmCas12m gene (F: 5′-ACCATGACAGTGCATACAATG-3′, R: 5′-CTCGATTCTGGCCTGCTTCA-3′), psgRNA-MmCas12m plasmid (F: 5′-TTCATATTTGCATATACGAT-3′, R: 5′-GTGTTTCGTCCTTTCCACAA-3′) and pLentiPack plasmid (F: 5′-CAGAGATCCAGTTTGGAAAG-3′, R: 5′-TTGCCACACAATCATCACCT-3′). Real-time PCR was performed on a QuantStudio 5 thermocycler (Thermo Fisher Scientific) in 20 μL of BioMaster HS-Taq PCR reaction mix (Biolabmix, Novosibirsk, Russia) containing 0.4 pM of each primer and 0.2 pM of a probe under the following conditions: 95 °C (5 min); 45 cycles of 95 °C (10 s), 57 °C (20 s), and 72 °C (20 s).

## 3. Results

### 3.1. Identifying the Base Editor with a Narrowed Editing Window

**Figure 1:**
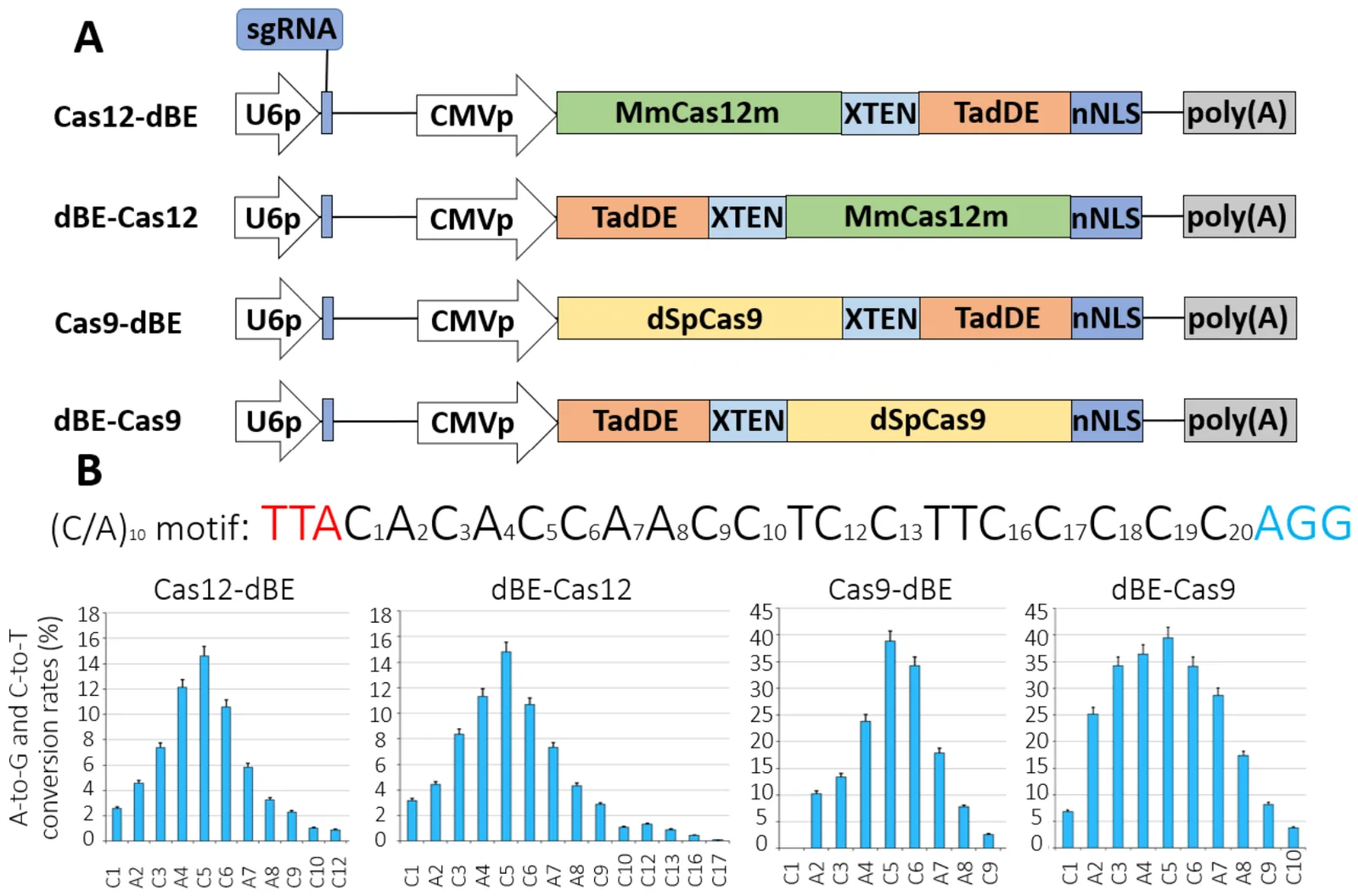
Comparison of N- and C-terminal deaminase fusions to MmCas12m and dSpCas9. (**A**) The image of the expression cassettes of pCas12-dBE, pCas9-dBE, pdBE-Cas12, and pdBE-Cas9the used in the experiment. (**B**) Transfection-mediated editing efficiency. The sequence of the target (C/A)_10_ motif is shown, with the numbers representing the position of possible editing targets relative to the MmCas12m PAM (red) and SpCas9 PAM (blue). The percentage of A-to-G and C-to-T editing represents the percentage of total sequencing reads with conversions. U6p–U6 promoter, sgRNA—single guide RNA, CMVp–cytomegalovirus promoter, MmCas12m—the MmCas12m domain, dSpCas9—the catalytically inactive version of the *Streptococcus pyogenes* Cas9 domain, TadDE—the dual deaminase based on TadA-8e, XTEN—the XTEN linker, and nNLS—the nuclear localization signal of nucleoplasmin. Values and error bars represent the mean and standard deviation of three independent biological replicates. Source data are provided in Supplementary Figure S9.

In the first step, to better understand what affects the width of the activity window, we designed and obtained four constructs for comparison of N- and C-terminal fusions of TadDE to MmCas12m and dSpCas9, initially using the XTEN linker (Figure 1A). The 16 aa flexible linker XTEN is a compromise between editing efficiency and specificity [13]. SpCas9 was selected as a comparison model to confirm editing only at the first stage. SpCas9 is well-annotated and widely used for base editing. We chose the (C/A)_10_ motif at the AAVS1 locus as the initial target to determine the width of the editing window. The (C/A)_10_ motif is flanked by the MmCas12m PAM and SpCas9 PAM (Figure 1). When the deaminase domain was fused to the N-terminus of MmCas12m and dSpCas9, the editing activity was similar (Figure 1B). We detected differences in the width of the editing window. The N-terminal TadDE resulted in editing in a much broader window when tested on the (C/A)_10_ motif. The C-terminal fusion showed a more specific editing activity, peaking at the N_5_ position within the protospacer (Figure 1). The editing efficiency has reached about 16% for MmCas12m-TadDE (Cas12-dBE for short in some cases) and 40% for dSpCas9-TadDE.

### 3.2. Optimization of the Linker Length

**Figure 2:**
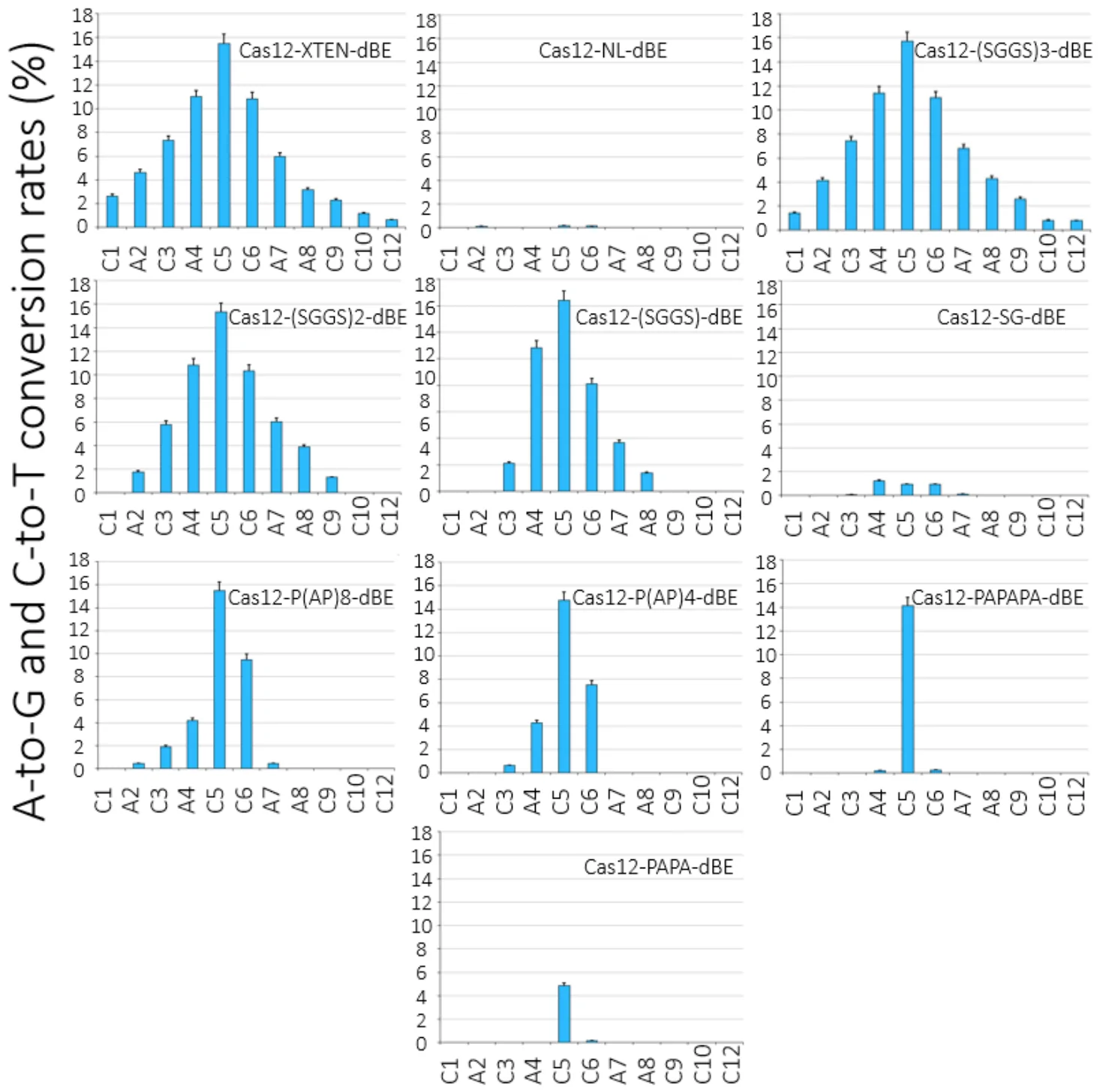
Comparison of different linkers. Editing efficiency and specificity of the base editors tested by the target (C/A)_10_ motif. The x-axis shows the target C/A within the protospacers. The y-axis shows editing frequency. NL—no linker. Values and error bars represent the mean and standard deviation of three independent biological replicates. Source data are provided in Supplementary Figure S10.

The Cas12-dBE was chosen as the basis for the future high-precision MmCas12m dual base editor. The choice is due to the fact that the Cas12-dBE showed a narrowed editing window with equal efficiency compared to the dBE-Cas12. Then, we varied the length of the linker between the domains of the Cas nuclease and deaminase. Using the (C/A)_10_ motif, we investigated the effects of length and rigidity of the linker between MmCas12m and TadDE on base editing precision and efficiency. We tested eight different linker sequences (containing the glycine–serine or amino acid proline that, due to its secondary amine, confers conformational rigidity) in comparison to the XTEN flexible linker. Consistent with the previous experiment, the base editor Cas12-XTEN-dBE (containing the XTEN linker) allowed editing at all C/A within a window of twelve nucleotides (Figure 2). The lack of the linker sequence or use of a very short linker precluded editing. A rigid proline linker of 6 aa (PAPAPA) made editing substantially more precise compared to others. Longer linkers lead to lower editing accuracy, suggesting that a seven aa rigid linker is optimal. The Cas12-PAPAPA-dBE showed improved site selectivity and a narrowed editing window, while retaining up to 90% of the editing efficiency of the original Cas12-XTEN-dBE.

### 3.3. Genome Editing by High-Precision MmCas12m Dual Base Editor-Loaded Virus-like Particles

Tests on the oligo(C/A)_10_ motif are the most stringent method for site selectivity of base editors. However, long (C/A) editing windows would rarely be targets of genome editing with base editors in vitro or in vivo. To test whether our high-precision Cas12-dBE also demonstrates performance in more heteropolymeric genomic sequence contexts, we targeted seven sites in the AAVS1 locus. Each site contains different sequence contexts with additional C/A directly adjacent to or close to the peak position (N_5_), or without it. In addition, in one case, the peak position is G (Target 3). VLPs are selected as the delivery platform. To generate VLP-MmCas12m-TadDE, we constructed a plasmid (pGag-MmCas12m-TadDE) that expresses a 6×His-tagged MmCas12m-TadDE fused to lentiviral structural proteins. The resulting VLPs were collected and concentrated roughly 50-fold via centrifugation at 21,000× *g* for 2.5 h, following the methodology outlined in Section 2 and reference [23]. We analyzed Cas-based complex packaging into particles using Western blot (Supplementary Figure S11).

**Figure 3:**
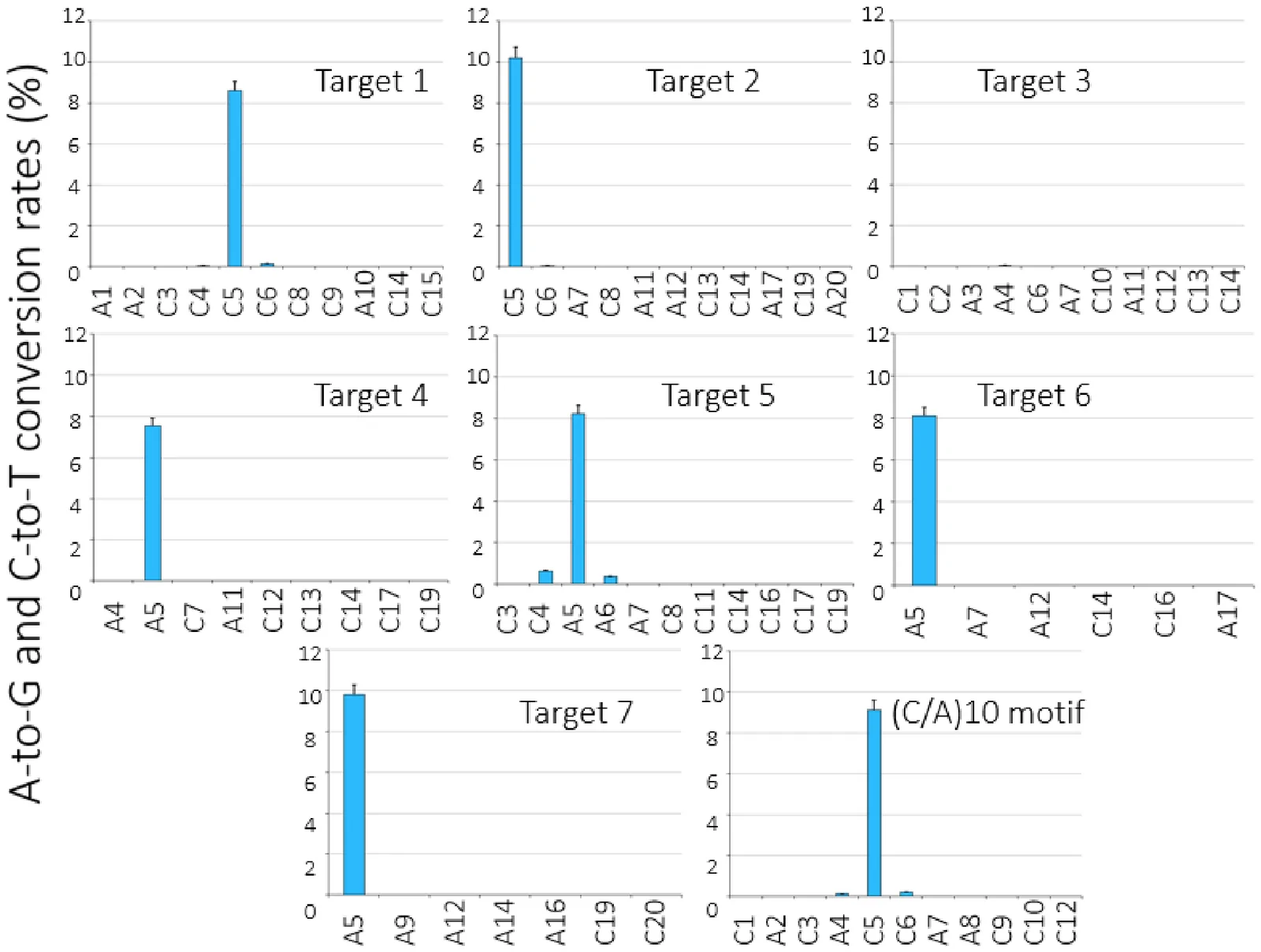
The efficiency of VLP-MmCas12m-TadDE-mediated editing of target sites in the AAVS1 locus. The sequence of the target C/A is shown, with the numbers representing the position of possible editing targets relative to the MmCas12m PAM. Values and error bars represent the mean and standard deviation of three independent biological replicates. Source data are provided in Supplementary Figure S12.

The HEK293T cells were treated with concentrated VLPs (100 µL dose). After 50 h of incubation, the cells of each group were collected, and genomic DNA was isolated. The isolated genomic DNA was used for amplification of PCR products. The obtained amplicons were analyzed using the NGS method (Figure 3).

The efficiency of VLP-mediated editing was lower than that of plasmid transfection-mediated editing. Adjacent nucleotides were edited with low efficiency (less than 0.8%). In one case, the adjacent adenine (Target 4) was not edited at all. In all cases, it was found that the C/A located one nucleotide away was not edited. We detected a decrease in the editing efficiency even in the oligo(C/A)_10_ motif. We assumed that the stoichiometry of original VLPs is probably suboptimal.

### 3.4. Improving Component Stoichiometry of VLP

**Figure 4:**
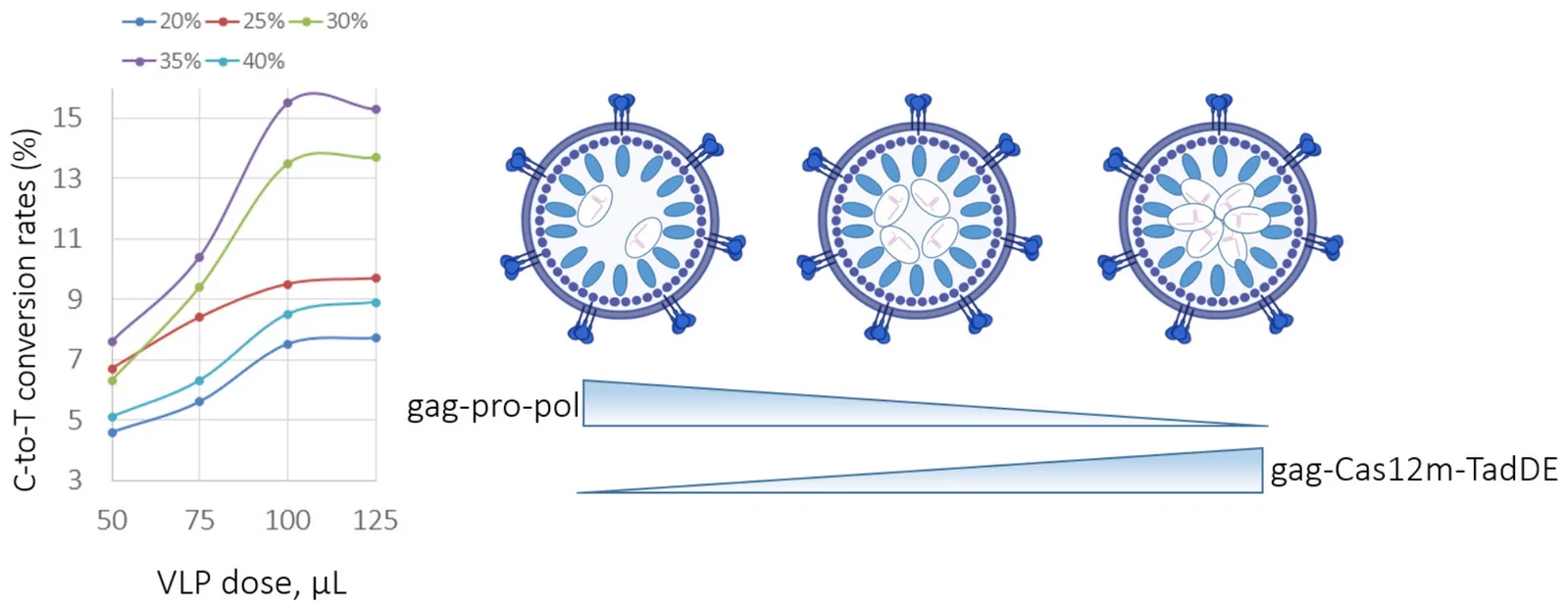
Cytosine base editing efficiency of VLP-MmCas12m-TadDE with different gag–pro–pol:gag–cargo stoichiometries at position C_5_ of the (C/A)_10_ motif in the AAVS1 locus of HEK293T cells. The x-axis shows the VLP dose. The y-axis shows editing frequency.

We optimized the gag–pro–pol:gag–cargo stoichiometry. Previous studies confirmed that an optimal gag–pro–pol:gag–cargo stoichiometry balance is the amount of gag–cargo available to be packaged into VLPs relative to the amount of lentiviral protease required for VLP maturation. To determine optimal stoichiometry, we varied the ratio of gag–MmCas12m-TadDE to wild-type LV gag–pro–pol plasmids transfected for VLP production (Figure 4).

We observed that increasing the proportion of the gag–MmCas12m-TadDE plasmid (up to 35%) beyond the original proportion used for production (25% of the gag–MmCas12m-TadDE plasmid and 75% of the wild-type gag–pro–pol plasmid) improved the editing efficiency. However, further increasing the amount of the gag–MmCas12m-TadDE plasmid to 40% led to a decrease in the editing efficiency. Decreasing the proportion of the gag–MmCas12m-TadDE plasmid from 25% to 20% reduced the editing efficiency too (Figure 4). The optimal dose for subsequent experiments was set to 100 µL, since an increase did not improve the editing efficiency.

The results of this VLP optimization revealed a VLP-MmCas12m-TadDE formulation that combines the optimal gag–pro–pol:gag–MmCas12m-TadDE stoichiometry. We visualized optimized VLPs by electron microscopy and confirmed their spherical morphology and an approximate particle diameter of 120–150 nm (Supplementary Figure S13).

### 3.5. Multi-Target Gene Editing via Original “Kombi” VLP

**Figure 5:**
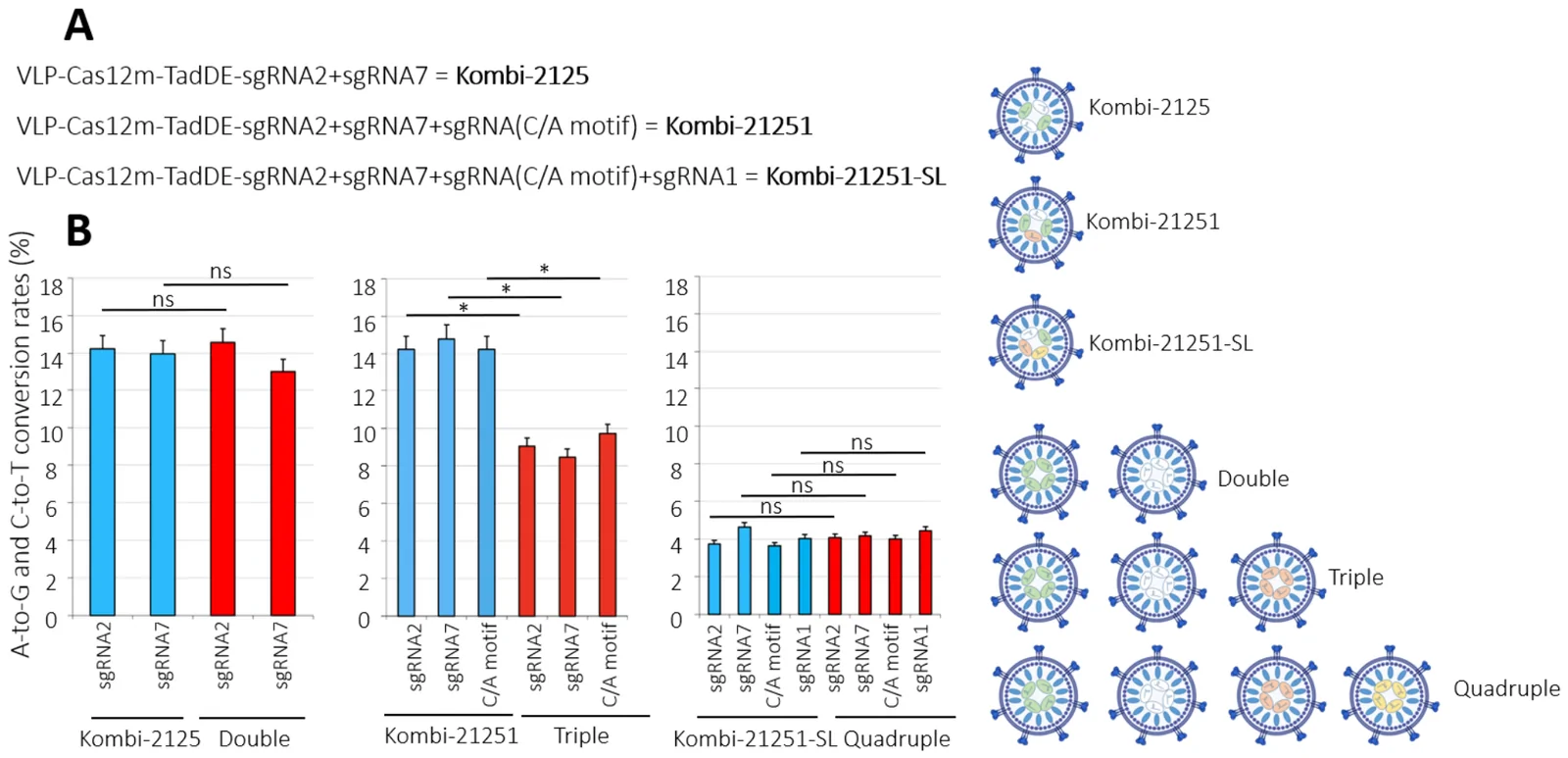
Multi-target editing. The combi approach involved packaging two or more sgRNAs into a single VLP. (**A**) VLP names for the combi method. (**B**) Base editing efficiency. The combi approach is blue. Double, triple, or quadruple methods are red. The x-axis shows the methods and sgRNAs used. The y-axis shows editing frequency. * *p* < 0.05 based on Tukey’s test for all comparisons. ns—not significant.

Previous research has demonstrated the feasibility of multi-target editing using VLPs carrying the CRISPR/Cas ribonucleoproteins. To assess the potential for multi-gene targeting, we performed editing in HEK293T cells using VLP-MmCas12m-TadDE via different delivery strategies (combi, double, triple, and quadruple). The combi method packages two, three, or four sgRNAs into a single VLP, simultaneously targeting two, three, or four loci, respectively. Conversely, in the double, triple, or quadruple methods, each sgRNA is packaged into a separate VLP for multi-targeting. We consistently combined sgRNA2, sgRNA7, the sgRNA(C/A)_10_ motif, and sgRNA1, as these demonstrated the highest editing efficiency in our previous experiments. We named VLPs for the combi method “Kombi”, followed by a specific numerical index for brevity (Figure 5A).

Kombi-2125 demonstrated a simultaneous two-locus editing efficiency comparable to that of the double method. Notably, Kombi-21251, which carries three sgRNAs, maintained the baseline editing efficiency at approximately 15%. In contrast, the triple method led to a decrease in editing efficiency. This reduction is likely due to an excess of VLPs in the suspension, which may induce cytotoxicity in the cells. However, this is just one possible explanation. Unexpectedly, the use of Kombi-21251-SL resulted in a threefold decrease in the editing efficiency. The fourth part of the plasmid encoding sgRNA is probably insufficient. Similarly, the quadruple method exhibited reduced editing performance, continuing this downward trend. Therefore, the combi method supports multi-target editing at an efficiency level comparable to, or even exceeding, that of the classic double, triple, or quadruple methods. No indels were detected within the editing windows during the analysis.

To detect residual plasmid DNA used for VLP production in the extracted genomic DNA samples, we performed real-time PCR using four primer pairs per plasmid. No plasmid DNA was detected, confirming that the treated HEK293T cells were free of exogenous DNA. To evaluate potential off-target effects, we used Cas-OFFinder software (v2.4.1) to predict off-target sites across the entire HEK293T genome, allowing for up to three mismatches relative to the on-target sequence. The predicted off-target loci are listed in Supplementary Figure S15. Following editing with “Kombi” VLPs, we analyzed these sites using NGS and found no de novo mutations. This off-target analysis was restricted to sgRNA1, sgRNA2, sgRNA7, and the sgRNA(C/A)_10_ motif.

## 4. Discussion

In this study, we have used the strategy to effectually narrow the editing window and thus improve the precision of the MmCas12m dual base editor. Indeed, the use of rigid, proline-rich linkers of a certain length can narrow the editing window and, thus, increase the accuracy of editing. A similar pattern was found in another study where CBE was used [19]. Perhaps the shortening of the linker leads to the constriction of the movements of the domains relative to each other. Thus, there is more precise positioning of the deaminase domain on the target site. The Cas12-dBE served as the basis for the high-precision MmCas12m dual base editor, because the Cas12-dBE showed a narrowed editing window with equal efficiency compared to the dBE-Cas12. Notably, N- and C-terminal fusions of the deaminase to the Cas domain lead to different editing efficiencies and variations in the editing window in other studies [15,19,27]. Usually, the greater efficiency and the broader editing window are observed in the case of N-terminal fusions of the deaminase to the Cas domain. Notably, C-terminal fusion of a deaminase, such as APOBEC1, to the Cas protein does not result in base editing. In contrast, N- and C-terminal fusions of the CDA1 deaminase to the Cas protein affect the width of the editing window, but not the peak editing efficiency [19]. N-terminal fusion of CDA1 to the Cas protein results in a broader editing window.

The developed MmCas12m dual base editor has a narrowed activity window. The MmCas12m dual base editor primarily edits at position (C/A)_5_ after PAM. Editing is successfully performed in both plasmid transfection and VLP treatment. At the same time, the efficiency was similar for both types of editing (about 15%). Maintaining the editing efficiency via VLP treatment has become possible due to the stoichiometry optimization. Apparently, VLPs require individual optimization of stoichiometry depending on cargo. In our case, the optimal percentage of plasmid expressing gag-cargo was 35%. In other studies, where prime editing tools were delivered via VLPs, this value was 25% [15]. The reason is probably related to the size of the cargo. MmCas12m-TadDE is smaller than the prime editing tool, so more of its molecules can be packed into the VLP.

The combi strategy looks promising. A single VLP that provides editing of several loci at once greatly facilitates the task of multi-target editing. A similar strategy was used earlier to combine different sgRNAs in a single combi VLP. The combi VLP has been successfully used for transgenesis of laboratory animals via treatment of zygotes [28]. However, this approach does not allow using different tools together (for example, Cas and the base editor), as neither tool can selectively distinguish between individual sgRNAs, which can lead to unintended mutations.

High-precision and compact base editors will certainly be needed for future genome editing applications. This approach will be extremely useful if it is necessary to edit a single-nucleotide polymorphism, and multiple mutagenesis is not desirable.

It is important to acknowledge several limitations of this study. First, the genome editing was evaluated exclusively in the HEK293T cell line. Second, whole-genome sequencing was not performed to assess potential off-target effects.

## 5. Conclusions

We have engineered a high-precision MmCas12m dual base editor that retains up to 90% of the editing efficiency of the original base editor by varying the length of the linker and the different locations of the domains relative to each other. We successfully delivered our base editor using single and combi VLPs to the loci of the HEK293T genome and confirmed editing with minimal bystander effect on neighboring nucleotides. Notably, editing accuracy and efficiency were evaluated exclusively at the AAVS1 locus.

## Figures and Tables

**Table 1:** **Plasmids used in this study.**

Plasmid Name	Description
pLentiPack	Lentiviral packaging plasmid
pCMV-VSV-G (#8454, Addgene)	Lentiviral envelope plasmid
pLenti CMV GFP Puro (#17448, Addgene)	Lentiviral transfer plasmid
dSpCas9 (#201953, Addgene)	Mammalian expression plasmid of catalytically inactive SpCas9
SpCas9_sgRNA (#122089, Addgene)	U6-driven sgRNA expression vector for cloning own guides
pCas12-dBE	Codes sgRNA and the TadDE fused to the C-terminus of MmCas12m
pdBE-Cas12	Codes sgRNA and the TadDE fused to the N-terminus of MmCas12m
pCas9-dBE	Codes sgRNA and the TadDE fused to the C-terminus of dSpCas9
pdBE-Cas9	Codes sgRNA and the TadDE fused to the N-terminus of dSpCas9
pGag-MmCas12m-TadDE	Codes VLP-MmCas12m-TadDE components fused with 6×His tag
psgRNA-MmCas12m	U6-driven MmCas12m sgRNA expression vector for cloning own guides for VLP production

## Data Availability

The original contributions presented in this study are included in the article/Supplementary Materials. Further inquiries can be directed to the corresponding authors.

## Supplementary Materials

The following supporting information can be downloaded at: https://www.mdpi.com/article/10.3390/genes17101247/s1; Figure S1: The complete nucleotide sequence coding for dBE-Cas12; Figure S2: The complete nucleotide sequence coding for Cas12-dBE; Figure S3: The complete nucleotide sequence coding for Cas9-dBE; Figure S4: The complete nucleotide sequence coding for dBE-Cas9; Figure S5: DNA fragments used to create pCas12-dBE, pdBE-Cas12, psgRNA-Cas12m (A), and pCas9-dBE, pdBE-Cas9 (B); Figure S6: List of linkers used in the Cas12-dBE constructs; Figure S7: The complete nucleotide sequence coding for the Gag-MmCas12m-TadDE-6xHis tag fusion protein; Figure S8: Primers used in this study to amplify target sites for NGS; Figure S9: Comparison of N- and C-terminal deaminase fusions to MmCas12m and dSpCas9. Source data; Figure S10: Comparison of different linkers. Editing efficiency and specificity of the base editors tested by the target (C/A)_10_ motif. Source data; Figure S11: Western blot confirming the content of MmCas12m-TadDE editing complexes (about 86 kDa) in VLP for each of the eight AAVS1 targets.; Figure S12: The efficiency of VLP-MmCas12m-mediated editing of target sites in the AAVS1 locus. Source data; Figure S13: The electron microscopic image of stoichiometrically improved Virus-like particles; Figure S14: Restriction digest of plasmids used for VLP production; Figure S15: The list of potential off-target sites during sgRNA1, 2, 7, and sgRNA(C/A)_10_-mediated editing.

## Abbreviations

The following abbreviations are used in this manuscript:

Cas	RNA-programmable CRISPR-associated nuclease
DSB	Double-stranded DNA break
NHEJ	Non-homologous end-joining
MMEJ	Microhomology-mediated end-joining
HDR	Homology-directed repair
SNP	Single-nucleotide polymorphism
BE	Base editor
CBE	Cytosine base editor
ABE	Adenine base editor
TadDE	Dual deaminase based on TadA-8e
dBE	Dual base editor
FBS	Fetal bovine serum
VLPs	Virus-like particles
sgRNA	Single guide RNA
PAM	Protospacer-adjacent motif
aa	Amino acid
AAV	Adeno-associated virus
AAVS1	Adeno-associated virus integration site 1
