# Supplementary material for: Engineering of the High-Precision Cas12m Dual Base Editor-Loaded Virus-like Particles for A-to-G and C-to-T Conversions
Source: Genes (Basel). 2026 Oct 9;17(10):1247. doi: 10.3390/genes17101247 (PMC13704335; doi:10.3390/genes17101247)
Supplement: Supplementary file 1 [file genes-17-01247-s001.zip › Supp figures_Genes.pdf]

ATGAGCGAAGTGGAATTCAGCCATGAATATTGGATGCGTCATGCACTGACCCTGGC
AAAACGTGCCCGTGATGAAGGTGAAGCACCGGTTGGTGCAGTTCTGGTTCTGAATA
ATCGTGTTATTGGTGAAGGTTGGAATCGTCGTATTGGTCTGCATGATCCGACCGCAC
ATGCAGAAATTATGGCACTGCGTCAAGGTGGTCTGGTTATGCAGAATAGCCGTCTGA
TTGATGCAACCCTGTATGTTACCTTTGAACCGTGTGTTATGTGTGCCGGTGCAATGAT
TAATAGCCGCATTGGTCGTGTTGTTTTTGGTGTTCGTAATAGCAAACGTGGTGCAGC
AGGTAGCCTGATGAATGTTCTGAATTATCCGGGTATGAATCACCGTGTTGAAATTAC
CGAAGGTATTCTGGCAGATGAATGTGCAGCACTGCTGTGTGATTTTTATCGTATGCC
TCGTCAGGTTTTTAACGCACAGAAAAAGGCACAGAGCAGCATTAATAGCGGCAGCG
AGACTCCCGGGACCTCAGAGTCCGCCACACCCGAAAGTACCACCATGACAGTGCAT
ACAATGGGCGTGCACTACAAGTGGCAGATCCCCGAGGTTCTGAGACAGCAGCTGTG
GCTGGCCCACAACCTGAGAGAAGATCTGGTGTCTCTGCAGCTGGCCTACGACGACG
ACCTGAAGGCCATCTGGTCTAGCTACCCTGATGTGGCCCAGGCCGAGGATACAATG
GCTGCCGCTGAAGCTGATGCCGTGGCTCTGTCTGAGAGAGTGAAGCAGGCCAGAAT
CGAGGCCAGAAGCAAGAAGATCAGCACCGAGCTGACCCAGCAGCTGAGGGACGCC
AAGAAGAGACTGAAGGACGCCAGACAGGCCAGACGGGATGCCATTGCCGTGGTCA
AAGATGATGCCGCCGAGCGGAGAAAGGCCAGATCTGATCAACTGGCCGCCGACCAG
AAAGCCCTGTACGGCCAGTACTGCAGAGATGGCGATCTGTACTGGGCCAGCTTCAA
CACCGTGCTGGACCACCACAAGACCGCCGTGAAGAGAATCGCCGCTCAGAGAGCCT
CTGGCAAGCCTGCCACACTGAGACACCACAGATTTGACGGCAGCGGCACAATCGCC
GTGCAACTCCAAAGACAAGCTGGCGCCCCTCCTCGGACACCTATGGTTCTTGCTGAT
GAGGCCGGCAAGTACCGGAACGTGCTGCACATTCCTGGCTGGACCGATCCTGATGT
GTGGGAGCAGATGACACGGTCCCAGTGCAGACAGTCTGGCAGAGTGACAGTGCGGA
TGAGATGCGGCAGCACAGATGGACAGCCCCAGTGGATCGATCTGCCTGTGCAGGTT
CACAGATGGCTGCCTGCCGACGCCGATATTACAGGCGCTGAGCTGGTGGTCACAAG
AGTGGCCGGAATCTACAGAGCCAAGCTGTGCGTGACCGCCAGGATCGGAGATACCG
AGCCTGTGACATCTGGCCCTACAGTGGCCCTGCATCTTGGCTGGCGATCTACCGAAG
AGGGAACAGCCGTGGCCACTTGGAGATCTGATGCCCCTCTGGACATCCCCTTCGGCC
TGAGAACAGTGATGAGAGTGGATGCTGCCGGCACCTCTGGCATCATTGTGGTGCCTG
CCACCATCGAGAGAAGGCTGACCCGGACAGAGAATATCGCCAGCAGCAGATCTCTG
GCCCTGGACGCCCTGAGAGATAAGGTTGTCGGCTGGCTGAGCGACAACGACGCCCC
TACCTATAGAGATGCTCCACTGGAAGCCGCCACCGTGAAGCAGTGGAAGTCCCCTC
AGAGATTCGCCTCTCTGGCTCACGCCTGGAAGGACAATGGCACCGAGATCAGCGAT
ATCCTGTGGGCCTGGTTCAGCCTGGACAGAAAGCAGTGGGCTCAGCAAGAGAACGG
CAGAAGAAAGGCCCTGGGACACAGAGATGACCTGTATCGGCAGATCGCCGCCGTGA
TCTCTGATCAGGCTGGACACGTGCTGGTGGACGATACAAGTGTGGCCGAGCTGAGC
GCCAGAGCTATGGAAAGAACCGAGCTGCCTACCGAGGTGCAGCAGAAGATCGACA
GAAGAAGGGACCACGCTGCTCCCGGTGGACTGAGAGCTTCTGTGGTGGCCGCCATG
ACAAGAGATGGGGTGCCAGTGACAATCGTGGCCGCAGCCGATTTCACCCGGACACA
CAGCAGATGCGGACACGTGAACCCCGCCGACGATAGATACCTGAGCAACCCCGTCA
GATGCGACGGCTGTGGCGCTATGTACGACCAGGACAGATCCTTCGTGACCCTGATGC
TGAGAGCCGCCACAGCTCCTAGCAATCCTGGCAAAAGACCTGCCGCCACAAAGAAG
GCCGGACAGGCCAAAAAGAAAAAG

Supplementary Figure S1. The complete nucleotide sequence coding for dBE-Cas12.

Orange = the TadDE, Violet = the XTEN linker, Black = the *Mycolicibacterium mucogenicum* Cas12m, Blue = nucleoplasmin NLS.

ATGACCACCATGACAGTGCATACAATGGGCGTGCACTACAAGTGGCAGATCCCCGA
GGTTCTGAGACAGCAGCTGTGGCTGGCCCACAACCTGAGAGAAGATCTGGTGTCTCT
GCAGCTGGCCTACGACGACGACCTGAAGGCCATCTGGTCTAGCTACCCTGATGTGGC
CCAGGCCGAGGATACAATGGCTGCCGCTGAAGCTGATGCCGTGGCTCTGTCTGAGA
GAGTGAAGCAGGCCAGAATCGAGGCCAGAAGCAAGAAGATCAGCACCGAGCTGAC
CCAGCAGCTGAGGGACGCCAAGAAGAGACTGAAGGACGCCAGACAGGCCAGACGG
GATGCCATTGCCGTGGTCAAAGATGATGCCGCCGAGCGGAGAAAGGCCAGATCTGA
TCAACTGGCCGCCGACCAGAAAGCCCTGTACGGCCAGTACTGCAGAGATGGCGATC
TGTACTGGGCCAGCTTCAACACCGTGCTGGACCACCACAAGACCGCCGTGAAGAGA
ATCGCCGCTCAGAGAGCCTCTGGCAAGCCTGCCACACTGAGACACCACAGATTTGA
CGGCAGCGGCACAATCGCCGTGCAACTCCAAAGACAAGCTGGCGCCCCTCCTCGGA
CACCTATGGTTCTTGCTGATGAGGCCGGCAAGTACCGGAACGTGCTGCACATTCCTG
GCTGGACCGATCCTGATGTGTGGGAGCAGATGACACGGTCCCAGTGCAGACAGTCT
GGCAGAGTGACAGTGCGGATGAGATGCGGCAGCACAGATGGACAGCCCCAGTGGA
TCGATCTGCCTGTGCAGGTTCACAGATGGCTGCCTGCCGACGCCGATATTACAGGCG
CTGAGCTGGTGGTCACAAGAGTGGCCGGAATCTACAGAGCCAAGCTGTGCGTGACC
GCCAGGATCGGAGATACCGAGCCTGTGACATCTGGCCCTACAGTGGCCCTGCATCTT
GGCTGGCGATCTACCGAAGAGGGAACAGCCGTGGCCACTTGGAGATCTGATGCCCC
TCTGGACATCCCCTTCGGCCTGAGAACAGTGATGAGAGTGGATGCTGCCGGCACCTC
TGGCATCATTGTGGTGCCTGCCACCATCGAGAGAAGGCTGACCCGGACAGAGAATA
TCGCCAGCAGCAGATCTCTGGCCCTGGACGCCCTGAGAGATAAGGTTGTCGGCTGG
CTGAGCGACAACGACGCCCCTACCTATAGAGATGCTCCACTGGAAGCCGCCACCGT
GAAGCAGTGGAAGTCCCCTCAGAGATTCGCCTCTCTGGCTCACGCCTGGAAGGACA
ATGGCACCGAGATCAGCGATATCCTGTGGGCCTGGTTCAGCCTGGACAGAAAGCAG
TGGGCTCAGCAAGAGAACGGCAGAAGAAAGGCCCTGGGACACAGAGATGACCTGT
ATCGGCAGATCGCCGCCGTGATCTCTGATCAGGCTGGACACGTGCTGGTGGACGAT
ACAAGTGTGGCCGAGCTGAGCGCCAGAGCTATGGAAAGAACCGAGCTGCCTACCGA
GGTGCAGCAGAAGATCGACAGAAGAAGGGACCACGCTGCTCCCGGTGGACTGAGA
GCTTCTGTGGTGGCCGCCATGACAAGAGATGGGGTGCCAGTGACAATCGTGGCCGC
AGCCGATTTCACCCGGACACACAGCAGATGCGGACACGTGAACCCCGCCGACGATA
GATACCTGAGCAACCCCGTCAGATGCGACGGCTGTGGCGCTATGTACGACCAGGAC
AGATCCTTCGTGACCCTGATGCTGAGAGCCGCCACAGCTCCTAGCAATCCTGGCAGC
GGCAGCGAGACTCCCGGGACCTCAGAGTCCGCCACACCCGAAAGTAGCGAAGTGGA
ATTCAGCCATGAATATTGGATGCGTCATGCACTGACCCTGGCAAAACGTGCCCGTGA
TGAAGGTGAAGCACCGGTTGGTGCAGTTCTGGTTCTGAATAATCGTGTTATTGGTGA
AGGTTGGAATCGTCGTATTGGTCTGCATGATCCGACCGCACATGCAGAAATTATGGC
ACTGCGTCAAGGTGGTCTGGTTATGCAGAATAGCCGTCTGATTGATGCAACCCTGTA
TGTTACCTTTGAACCGTGTGTTATGTGTGCCGGTGCAATGATTAATAGCCGCATTGG
TCGTGTTGTTTTTGGTGTTCGTAATAGCAAACGTGGTGCAGCAGGTAGCCTGATGAA
TGTTCTGAATTATCCGGGTATGAATCACCGTGTTGAAATTACCGAAGGTATTCTGGC
AGATGAATGTGCAGCACTGCTGTGTGATTTTTATCGTATGCCTCGTCAGGTTTTTAAC
GCACAGAAAAAGGCACAGAGCAGCATTAATAAAAGACCTGCCGCCACAAAGAAGG
CCGGACAGGCCAAAAAGAAAAAG

Supplementary Figure S2. The complete nucleotide sequence coding for Cas12-dBE.

Black = the *Mycolicibacterium mucogenicum* Cas12m, Violet = the XTEN linker, Orange = the TadDE, Blue = nucleoplasmin NLS.

ATGGACAAGAAGTACAGCATCGGCCTGGCCATCGGCACCAACTCTGTGGGCTGGGC
CGTGATCACCGACGAGTACAAGGTGCCCAGCAAGAAATTCAAGGTGCTGGGCAACA
CCGACCGGCACAGCATCAAGAAGAACCTGATCGGAGCCCTGCTGTTCGACAGCGGC
GAAACAGCCGAGGCCACCCGGCTGAAGAGAACCGCCAGAAGAAGATACACCAGAC
GGAAGAACCGGATCTGCTATCTGCAAGAGATCTTCAGCAACGAGATGGCCAAGGTG
GACGACAGCTTCTTCCACAGACTGGAAGAGTCCTTCCTGGTGGAAGAGGATAAGAA
GCACGAGCGGCACCCCATCTTCGGCAACATCGTGGACGAGGTGGCCTACCACGAGA
AGTACCCCACCATCTACCACCTGAGAAAGAAACTGGTGGACAGCACCGACAAGGCC
GACCTGCGGCTGATCTATCTGGCCCTGGCCCACATGATCAAGTTCCGGGGCCACTTC
CTGATCGAGGGCGACCTGAACCCCGACAACAGCGACGTGGACAAGCTGTTCATCCA
GCTGGTGCAGACCTACAACCAGCTGTTCGAGGAAAACCCCATCAACGCCAGCGGCG
TGGACGCCAAGGCCATCCTGTCTGCCAGACTGAGCAAGAGCAGACGGCTGGAAAAT
CTGATCGCCCAGCTGCCCGGCGAGAAGAAGAATGGCCTGTTCGGAAACCTGATTGC
CCTGAGCCTGGGCCTGACCCCCAACTTCAAGAGCAACTTCGACCTGGCCGAGGATG
CCAAACTGCAGCTGAGCAAGGACACCTACGACGACGACCTGGACAACCTGCTGGCC
CAGATCGGCGACCAGTACGCCGACCTGTTTCTGGCCGCCAAGAACCTGTCCGACGCC
ATCCTGCTGAGCGACATCCTGAGAGTGAACACCGAGATCACCAAGGCCCCCCTGAG
CGCCTCTATGATCAAGAGATACGACGAGCACCACCAGGACCTGACCCTGCTGAAAG
CTCTCGTGCGGCAGCAGCTGCCTGAGAAGTACAAAGAGATTTTCTTCGACCAGAGC
AAGAACGGCTACGCCGGCTACATTGACGGCGGAGCCAGCCAGGAAGAGTTCTACAA
GTTCATCAAGCCCATCCTGGAAAAGATGGACGGCACCGAGGAACTGCTCGTGAAGC
TGAACAGAGAGGACCTGCTGCGGAAGCAGCGGACCTTCGACAACGGCAGCATCCCC
CACCAGATCCACCTGGGAGAGCTGCACGCCATTCTGCGGCGGCAGGAAGATTTTTA
CCCATTCCTGAAGGACAACCGGGAAAAGATCGAGAAGATCCTGACCTTCCGCATCC
CCTACTACGTGGGCCCTCTGGCCAGGGGAAACAGCAGATTCGCCTGGATGACCAGA
AAGAGCGAGGAAACCATCACCCCCTGGAACTTCGAGGAAGTGGTGGACAAGGGCG
CTTCCGCCCAGAGCTTCATCGAGCGGATGACCAACTTCGATAAGAACCTGCCCAACG
AGAAGGTGCTGCCCAAGCACAGCCTGCTGTACGAGTACTTCACCGTGTATAACGAG
CTGACCAAAGTGAAATACGTGACCGAGGGAATGAGAAAGCCCGCCTTCCTGAGCGG
CGAGCAGAAAAAGGCCATCGTGGACCTGCTGTTCAAGACCAACCGGAAAGTGACCG
TGAAGCAGCTGAAAGAGGACTACTTCAAGAAAATCGAGTGCTTCGACTCCGTGGAA
ATCTCCGGCGTGGAAGATCGGTTCAACGCCTCCCTGGGCACATACCACGATCTGCTG
AAAATTATCAAGGACAAGGACTTCCTGGACAATGAGGAAAACGAGGACATTCTGGA
AGATATCGTGCTGACCCTGACACTGTTTGAGGACAGAGAGATGATCGAGGAACGGC
TGAAAACCTATGCCCACCTGTTCGACGACAAAGTGATGAAGCAGCTGAAGCGGCGG
AGATACACCGGCTGGGGCAGGCTGAGCCGGAAGCTGATCAACGGCATCCGGGACAA
GCAGTCCGGCAAGACAATCCTGGATTTCCTGAAGTCCGACGGCTTCGCCAACAGAA
ACTTCATGCAGCTGATCCACGACGACAGCCTGACCTTTAAAGAGGACATCCAGAAA
GCCCAGGTGTCCGGCCAGGGCGATAGCCTGCACGAGCACATTGCCAATCTGGCCGG
CAGCCCCGCCATTAAGAAGGGCATCCTGCAGACAGTGAAGGTGGTGGACGAGCTCG
TGAAAGTGATGGGCCGGCACAAGCCCGAGAACATCGTGATCGAAATGGCCAGAGA
GAACCAGACCACCCAGAAGGGACAGAAGAACAGCCGCGAGAGAATGAAGCGGATC
GAAGAGGGCATCAAAGAGCTGGGCAGCCAGATCCTGAAAGAACACCCCGTGGAAA
ACACCCAGCTGCAGAACGAGAAGCTGTACCTGTACTACCTGCAGAATGGGCGGGAT
ATGTACGTGGACCAGGAACTGGACATCAACCGGCTGTCCGACTACGATGTGGACGC

CATCGTGCCTCAGAGCTTTCTGAAGGACGACTCCATCGACAACAAGGTGCTGACCA
GAAGCGACAAGAACCGGGGCAAGAGCGACAACGTGCCCTCCGAAGAGGTCGTGAA
GAAGATGAAGAACTACTGGCGGCAGCTGCTGAACGCCAAGCTGATTACCCAGAGAA
AGTTCGACAATCTGACCAAGGCCGAGAGAGGCGGCCTGAGCGAACTGGATAAGGCC
GGCTTCATCAAGAGACAGCTGGTGGAAACCCGGCAGATCACAAAGCACGTGGCACA
GATCCTGGACTCCCGGATGAACACTAAGTACGACGAGAATGACAAGCTGATCCGGG
AAGTGAAAGTGATCACCCTGAAGTCCAAGCTGGTGTCCGATTTCCGGAAGGATTTCC
AGTTTTACAAAGTGCGCGAGATCAACAACTACCACCACGCCCACGACGCCTACCTG
AACGCCGTCGTGGGAACCGCCCTGATCAAAAAGTACCCTAAGCTGGAAAGCGAGTT
CGTGTACGGCGACTACAAGGTGTACGACGTGCGGAAGATGATCGCCAAGAGCGAGC
AGGAAATCGGCAAGGCTACCGCCAAGTACTTCTTCTACAGCAACATCATGAACTTTT
TCAAGACCGAGATTACCCTGGCCAACGGCGAGATCCGGAAGCGGCCTCTGATCGAG
ACAAACGGCGAAACCGGGGAGATCGTGTGGGATAAGGGCCGGGATTTTGCCACCGT
GCGGAAAGTGCTGAGCATGCCCCAAGTGAATATCGTGAAAAAGACCGAGGTGCAGA
CAGGCGGCTTCAGCAAAGAGTCTATCCTGCCCAAGAGGAACAGCGATAAGCTGATC
GCCAGAAAGAAGGACTGGGACCCTAAGAAGTACGGCGGCTTCGACAGCCCCACCGT
GGCCTATTCTGTGCTGGTGGTGGCCAAAGTGGAAAAGGGCAAGTCCAAGAAACTGA
AGAGTGTGAAAGAGCTGCTGGGGATCACCATCATGGAAAGAAGCAGCTTCGAGAAG
AATCCCATCGACTTTCTGGAAGCCAAGGGCTACAAAGAAGTGAAAAAGGACCTGAT
CATCAAGCTGCCTGAGTACTCCCTGTTCGAGCTGGAAAACGGCCGGAAGAGAATGC
TGGCCTCTGCCGGCGAACTGCAGAAGGGAAACGAACTGGCCCTGCCCTCCAAATAT
GTGAACTTCCTGTACCTGGCCAGCCACTATGAGAAGCTGAAGGGCTCCCCCGAGGA
TAATGAGCAGAAACAGCTGTTTGTGGAACAGCACAAGCACTACCTGGACGAGATCA
TCGAGCAGATCAGCGAGTTCTCCAAGAGAGTGATCCTGGCCGACGCTAATCTGGAC
AAAGTGCTGTCCGCCTACAACAAGCACCGGGATAAGCCCATCAGAGAGCAGGCCGA
GAATATCATCCACCTGTTTACCCTGACCAATCTGGGAGCCCCTGCCGCCTTCAAGTA
CTTTGACACCACCATCGACCGGAAGAGGTACACCAGCACCAAAGAGGTGCTGGACG
CCACCCTGATCCACCAGAGCATCACCGGCCTGTACGAGACACGGATCGACCTGTCTC
AGCTGGGAGGCGACAGCGGCAGCGAGACTCCCGGGACCTCAGAGTCCGCCACACCC
GAAAGTAGCGAAGTGGAATTCAGCCATGAATATTGGATGCGTCATGCACTGACCCT
GGCAAAACGTGCCCGTGATGAAGGTGAAGCACCGGTTGGTGCAGTTCTGGTTCTGA
ATAATCGTGTTATTGGTGAAGGTTGGAATCGTCGTATTGGTCTGCATGATCCGACCG
CACATGCAGAAATTATGGCACTGCGTCAAGGTGGTCTGGTTATGCAGAATAGCCGTC
TGATTGATGCAACCCTGTATGTTACCTTTGAACCGTGTGTTATGTGTGCCGGTGCAAT
GATTAATAGCCGCATTGGTCGTGTTGTTTTTGGTGTTCGTAATAGCAAACGTGGTGC
AGCAGGTAGCCTGATGAATGTTCTGAATTATCCGGGTATGAATCACCGTGTTGAAAT
TACCGAAGGTATTCTGGCAGATGAATGTGCAGCACTGCTGTGTGATTTTTATCGTAT
GCCTCGTCAGGTTTTTAACGCACAGAAAAAGGCACAGAGCAGCATTAATAAAAGAC
CTGCCGCCACAAAGAAGGCCGGACAGGCCAAAAAGAAAAAG

Supplementary Figure S3. The complete nucleotide sequence coding for Cas9-dBE.

Black = the catalytically inactive *Streptococcus pyogenes* Cas9, Violet = the XTEN linker, Orange = the TadDE, Blue = nucleoplasmin NLS.

ATGAGCGAAGTGGAATTCAGCCATGAATATTGGATGCGTCATGCACTGACCCTGGC
AAAACGTGCCCGTGATGAAGGTGAAGCACCGGTTGGTGCAGTTCTGGTTCTGAATA
ATCGTGTTATTGGTGAAGGTTGGAATCGTCGTATTGGTCTGCATGATCCGACCGCAC
ATGCAGAAATTATGGCACTGCGTCAAGGTGGTCTGGTTATGCAGAATAGCCGTCTGA
TTGATGCAACCCTGTATGTTACCTTTGAACCGTGTGTTATGTGTGCCGGTGCAATGAT
TAATAGCCGCATTGGTCGTGTTGTTTTTGGTGTTCGTAATAGCAAACGTGGTGCAGC
AGGTAGCCTGATGAATGTTCTGAATTATCCGGGTATGAATCACCGTGTTGAAATTAC
CGAAGGTATTCTGGCAGATGAATGTGCAGCACTGCTGTGTGATTTTTATCGTATGCC
TCGTCAGGTTTTTAACGCACAGAAAAAGGCACAGAGCAGCATTAATAGCGGCAGCG
AGACTCCCGGGACCTCAGAGTCCGCCACACCCGAAAGTGACAAGAAGTACAGCATC
GGCCTGGCCATCGGCACCAACTCTGTGGGCTGGGCCGTGATCACCGACGAGTACAA
GGTGCCCAGCAAGAAATTCAAGGTGCTGGGCAACACCGACCGGCACAGCATCAAGA
AGAACCTGATCGGAGCCCTGCTGTTCGACAGCGGCGAAACAGCCGAGGCCACCCGG
CTGAAGAGAACCGCCAGAAGAAGATACACCAGACGGAAGAACCGGATCTGCTATCT
GCAAGAGATCTTCAGCAACGAGATGGCCAAGGTGGACGACAGCTTCTTCCACAGAC
TGGAAGAGTCCTTCCTGGTGGAAGAGGATAAGAAGCACGAGCGGCACCCCATCTTC
GGCAACATCGTGGACGAGGTGGCCTACCACGAGAAGTACCCCACCATCTACCACCT
GAGAAAGAAACTGGTGGACAGCACCGACAAGGCCGACCTGCGGCTGATCTATCTGG
CCCTGGCCCACATGATCAAGTTCCGGGGCCACTTCCTGATCGAGGGCGACCTGAACC
CCGACAACAGCGACGTGGACAAGCTGTTCATCCAGCTGGTGCAGACCTACAACCAG
CTGTTCGAGGAAAACCCCATCAACGCCAGCGGCGTGGACGCCAAGGCCATCCTGTC
TGCCAGACTGAGCAAGAGCAGACGGCTGGAAAATCTGATCGCCCAGCTGCCCGGCG
AGAAGAAGAATGGCCTGTTCGGAAACCTGATTGCCCTGAGCCTGGGCCTGACCCCC
AACTTCAAGAGCAACTTCGACCTGGCCGAGGATGCCAAACTGCAGCTGAGCAAGGA
CACCTACGACGACGACCTGGACAACCTGCTGGCCCAGATCGGCGACCAGTACGCCG
ACCTGTTTCTGGCCGCCAAGAACCTGTCCGACGCCATCCTGCTGAGCGACATCCTGA
GAGTGAACACCGAGATCACCAAGGCCCCCCTGAGCGCCTCTATGATCAAGAGATAC
GACGAGCACCACCAGGACCTGACCCTGCTGAAAGCTCTCGTGCGGCAGCAGCTGCC
TGAGAAGTACAAAGAGATTTTCTTCGACCAGAGCAAGAACGGCTACGCCGGCTACA
TTGACGGCGGAGCCAGCCAGGAAGAGTTCTACAAGTTCATCAAGCCCATCCTGGAA
AAGATGGACGGCACCGAGGAACTGCTCGTGAAGCTGAACAGAGAGGACCTGCTGCG
GAAGCAGCGGACCTTCGACAACGGCAGCATCCCCCACCAGATCCACCTGGGAGAGC
TGCACGCCATTCTGCGGCGGCAGGAAGATTTTTACCCATTCCTGAAGGACAACCGGG
AAAAGATCGAGAAGATCCTGACCTTCCGCATCCCCTACTACGTGGGCCCTCTGGCCA
GGGGAAACAGCAGATTCGCCTGGATGACCAGAAAGAGCGAGGAAACCATCACCCC
CTGGAACTTCGAGGAAGTGGTGGACAAGGGCGCTTCCGCCCAGAGCTTCATCGAGC
GGATGACCAACTTCGATAAGAACCTGCCCAACGAGAAGGTGCTGCCCAAGCACAGC
CTGCTGTACGAGTACTTCACCGTGTATAACGAGCTGACCAAAGTGAAATACGTGACC
GAGGGAATGAGAAAGCCCGCCTTCCTGAGCGGCGAGCAGAAAAAGGCCATCGTGG
ACCTGCTGTTCAAGACCAACCGGAAAGTGACCGTGAAGCAGCTGAAAGAGGACTAC
TTCAAGAAAATCGAGTGCTTCGACTCCGTGGAAATCTCCGGCGTGGAAGATCGGTTC
AACGCCTCCCTGGGCACATACCACGATCTGCTGAAAATTATCAAGGACAAGGACTT
CCTGGACAATGAGGAAAACGAGGACATTCTGGAAGATATCGTGCTGACCCTGACAC
TGTTTGAGGACAGAGAGATGATCGAGGAACGGCTGAAAACCTATGCCCACCTGTTC
GACGACAAAGTGATGAAGCAGCTGAAGCGGCGGAGATACACCGGCTGGGGCAGGC
TGAGCCGGAAGCTGATCAACGGCATCCGGGACAAGCAGTCCGGCAAGACAATCCTG
GATTTCCTGAAGTCCGACGGCTTCGCCAACAGAAACTTCATGCAGCTGATCCACGAC

GACAGCCTGACCTTTAAAGAGGACATCCAGAAAGCCCAGGTGTCCGGCCAGGGCGA
TAGCCTGCACGAGCACATTGCCAATCTGGCCGGCAGCCCCGCCATTAAGAAGGGCA
TCCTGCAGACAGTGAAGGTGGTGGACGAGCTCGTGAAAGTGATGGGCCGGCACAAG
CCCGAGAACATCGTGATCGAAATGGCCAGAGAGAACCAGACCACCCAGAAGGGAC
AGAAGAACAGCCGCGAGAGAATGAAGCGGATCGAAGAGGGCATCAAAGAGCTGGG
CAGCCAGATCCTGAAAGAACACCCCGTGGAAAACACCCAGCTGCAGAACGAGAAG
CTGTACCTGTACTACCTGCAGAATGGGCGGGATATGTACGTGGACCAGGAACTGGA
CATCAACCGGCTGTCCGACTACGATGTGGACGCCATCGTGCCTCAGAGCTTTCTGAA
GGACGACTCCATCGACAACAAGGTGCTGACCAGAAGCGACAAGAACCGGGGCAAG
AGCGACAACGTGCCCTCCGAAGAGGTCGTGAAGAAGATGAAGAACTACTGGCGGCA
GCTGCTGAACGCCAAGCTGATTACCCAGAGAAAGTTCGACAATCTGACCAAGGCCG
AGAGAGGCGGCCTGAGCGAACTGGATAAGGCCGGCTTCATCAAGAGACAGCTGGTG
GAAACCCGGCAGATCACAAAGCACGTGGCACAGATCCTGGACTCCCGGATGAACAC
TAAGTACGACGAGAATGACAAGCTGATCCGGGAAGTGAAAGTGATCACCCTGAAGT
CCAAGCTGGTGTCCGATTTCCGGAAGGATTTCCAGTTTTACAAAGTGCGCGAGATCA
ACAACTACCACCACGCCCACGACGCCTACCTGAACGCCGTCGTGGGAACCGCCCTG
ATCAAAAAGTACCCTAAGCTGGAAAGCGAGTTCGTGTACGGCGACTACAAGGTGTA
CGACGTGCGGAAGATGATCGCCAAGAGCGAGCAGGAAATCGGCAAGGCTACCGCC
AAGTACTTCTTCTACAGCAACATCATGAACTTTTTCAAGACCGAGATTACCCTGGCC
AACGGCGAGATCCGGAAGCGGCCTCTGATCGAGACAAACGGCGAAACCGGGGAGA
TCGTGTGGGATAAGGGCCGGGATTTTGCCACCGTGCGGAAAGTGCTGAGCATGCCC
CAAGTGAATATCGTGAAAAAGACCGAGGTGCAGACAGGCGGCTTCAGCAAAGAGTC
TATCCTGCCCAAGAGGAACAGCGATAAGCTGATCGCCAGAAAGAAGGACTGGGACC
CTAAGAAGTACGGCGGCTTCGACAGCCCCACCGTGGCCTATTCTGTGCTGGTGGTGG
CCAAAGTGGAAAAGGGCAAGTCCAAGAAACTGAAGAGTGTGAAAGAGCTGCTGGG
GATCACCATCATGGAAAGAAGCAGCTTCGAGAAGAATCCCATCGACTTTCTGGAAG
CCAAGGGCTACAAAGAAGTGAAAAAGGACCTGATCATCAAGCTGCCTGAGTACTCC
CTGTTCGAGCTGGAAAACGGCCGGAAGAGAATGCTGGCCTCTGCCGGCGAACTGCA
GAAGGGAAACGAACTGGCCCTGCCCTCCAAATATGTGAACTTCCTGTACCTGGCCA
GCCACTATGAGAAGCTGAAGGGCTCCCCCGAGGATAATGAGCAGAAACAGCTGTTT
GTGGAACAGCACAAGCACTACCTGGACGAGATCATCGAGCAGATCAGCGAGTTCTC
CAAGAGAGTGATCCTGGCCGACGCTAATCTGGACAAAGTGCTGTCCGCCTACAACA
AGCACCGGGATAAGCCCATCAGAGAGCAGGCCGAGAATATCATCCACCTGTTTACC
CTGACCAATCTGGGAGCCCCTGCCGCCTTCAAGTACTTTGACACCACCATCGACCGG
AAGAGGTACACCAGCACCAAAGAGGTGCTGGACGCCACCCTGATCCACCAGAGCAT
CACCGGCCTGTACGAGACACGGATCGACCTGTCTCAGCTGGGAGGCGACAAAAGAC
CTGCCGCCACAAAGAAGGCCGGACAGGCCAAAAAGAAAAAG

Supplementary Figure S4. The complete nucleotide sequence coding for dBE-Cas9.

Orange = the TadDE, Violet = the XTEN linker, Black = the catalytically inactive *Streptococcus pyogenes* Cas9, Blue = nucleoplasmin NLS.

A

GAGGGCCTATTTCCCATGATTCCTTCATATTTGCATATACGATACAAGGCTGTTAGA
GAGATAATTGGAATTAATTTGACTGTAAACACAAAGATATTAGTACAAAATACGTG
ACGTAGAAAGTAATAATTTCTTGGGTAGTTTGCAGTTTTAAAATTATGTTTTAAAAT
GGACTATCATATGCTTACCGTAACTTGAAAGTATTTCGATTTCTTGGCTTTATATATC
TTGTGGAAAGGACGAAACACCGTGTCATAGCCCAGCTTGGCGGGCGAAGGCCAAGA
CGGAGACCGACACAAGCTTCAGTGGTCTCTTTTTTTTT

B

GAGGGCCTATTTCCCATGATTCCTTCATATTTGCATATACGATACAAGGCTGTTAGA
GAGATAATTGGAATTAATTTGACTGTAAACACAAAGATATTAGTACAAAATACGTG
ACGTAGAAAGTAATAATTTCTTGGGTAGTTTGCAGTTTTAAAATTATGTTTTAAAAT
GGACTATCATATGCTTACCGTAACTTGAAAGTATTTCGATTTCTTGGCTTTATATATC
TTGTGGAAAGGACGAAACACCGGAGACCGACACAAGCTTCAGTGGTCTCTGTTTAA
GAGCTATGCTGGAAACAGCATAGCAAGTTTAAATAAGGCTAGTCCGTTATCAACTTG
AAAAAGTGGCACCGAGTCGGTGC

Supplementary Figure S5. DNA fragments used to create pCas12-dBE, pdBE-Cas12, psgRNA-Cas12m (A), and pCas9-dBE, pdBE-Cas9 (B).

Black = U6 promoter, Violet = RNA scaffold for MmCas12m, Blue = RNA scaffold for SpCas9, Red = the fragment with BsaI cloning sites.

Linker	Number of amino acids	Nucleotide sequence
NL (no linker)	0	-
XTEN	16	AGCGGCAGCGAGACTCCCGGGACCTCAGAGTCCGCCACACCCGAAAGT
$(SGGS)_3$	12	AGCGGTGGTAGCAGCGGTGGTAGCAGCGGTGGTAGC
$(SGGS)_2$	8	AGCGGTGGTAGCAGCGGTGGTAGC
(SGGS)	4	AGCGGTGGTAGC
SG	2	AGCGGT
$P(AP)_8$	17	CCCGCCCCAGCCCCAGCCCCAGCCCCAGCCCCAGCCCCAGCCCCAGCCCCA
$P(AP)_4$	9	CCCGCCCCAGCCCCAGCCCCAGCCCCA
PAPAPA	6	CCAGCCCCAGCCCCAGCC
PAPA	4	CCAGCCCCAGCC

Supplementary Figure S6. List of linkers used in the Cas12-dBE constructs.

ATGGGTGCGAGAGCGTCAGTATTAAGCGGGGGAGAATTAGATCGATGGGAAAAAAT
TCGGTTAAGGCCAGGGGGAAAGAAAAAATATAAATTAAAACATATAGTATGGGCAA
GCAGGGAGCTAGAACGATTCGCAGTTAATCCTGGCCTGTTAGAAACATCAGAAGGC
TGTAGACAAATACTGGGACAGCTACAACCATCCCTTCAGACAGGATCAGAAGAACT
TAGATCATTATATAATACAGTAGCAACCCTCTATTGTGTGCATCAAAGGATAGAGAT
AAAAGACACCAAGGAAGCTTTAGACAAGATAGAGGAAGAGCAAAACAAAAGTAAG
AAAAAAGCACAGCAAGCAGCAGCTGACACAGGACACAGCAATCAGGTCAGCCAAA
ATTACCCTATAGTGCAGAACATCCAGGGGCAAATGGTACATCAGGCCATATCACCT
AGAACTTTAAATGCATGGGTAAAAGTAGTAGAAGAGAAGGCTTTCAGCCCAGAAGT
GATACCCATGTTTTCAGCATTATCAGAAGGAGCCACCCCACAAGATTTAAACACCAT
GCTAAACACAGTGGGGGGACATCAAGCAGCCATGCAAATGTTAAAAGAGACCATCA
ATGAGGAAGCTGCAGAATGGGATAGAGTGCATCCAGTGCATGCAGGGCCTATTGCA
CCAGGCCAGATGAGAGAACCAAGGGGAAGTGACATAGCAGGAACTACTAGTACCCT
TCAGGAACAAATAGGATGGATGACACATAATCCACCTATCCCAGTAGGAGAAATCT
ATAAAAGATGGATAATCCTGGGATTAAATAAAATAGTAAGAATGTATAGCCCTACC
AGCATTCTGGACATAAGACAAGGACCAAAGGAACCCTTTAGAGACTATGTAGACCG
ATTCTATAAAACTCTAAGAGCCGAGCAAGCTTCACAAGAGGTAAAAAATTGGATGA
CAGAAACCTTGTTGGTCCAAAATGCGAACCCAGATTGTAAGACTATTTTAAAAGCAT
TGGGACCAGGAGCGACACTAGAAGAAATGATGACAGCATGTCAGGGAGTGGGGGG
ACCCGGCCATAAAGCAAGAGTTTTGGCTGAAGCAATGAGCCAAGTAACAAATCCAG
CTACCATAATGATACAGAAAGGCAATTTTAGGAACCAAAGAAAGACTGTTAAGTGT
TTCAATTGTGGCAAAGAAGGGCACATAGCCAAAAATTGCAGGGCCCCTAGGAAAAA
GGGCTGTTGGAAATGTGGAAAGGAAGGACACCAAATGAAAGATTGTACTGAGAGA
CAGGCTAATTTTTTAGGGAAGATCTGGCCTTCCCACAAGGGAAGGCCAGGGAATTTT
CTTCAGAGCAGACCAGAGCCAACAGCCCCACCAGAAGAGAGCTTCAGGTTTGGGGA
AGAGACAACAACTCCCTCTCAGAAGCAGGAGCCGATAGACAAGGAACTGTATCCTT
TAGCTTCCCTCAGATCACTCTTTGGCAGCGACCCCTCGTCACAATAAAGATAGGGGG
GCAATTAAAGGAAGCTCTATTAGATACAGGAGCAGATGATACAGTATTAGAAGAAA
TGAATTTGCCAGGAAGATGGAAACCAAAAATGATAGGGGGAATTGGAGGTTTTATC
AAAGTAAGACAGTATGATCAGATACTCATAGAAATCTGCGGACATAAAGCTATAGG
TACAGTATTAGTAGGACCTACACCTGTCAACATAATTGGAAGAAATCTGTTGACTCA
GATTGGCTGCACTTTAAATTTTCCCATTAGTCCTGGGGGCCCAGCGGCACCACCATG
ACAGTGCATACAATGGGCGTGCACTACAAGTGGCAGATCCCCGAGGTTCTGAGACA
GCAGCTGTGGCTGGCCCACAACCTGAGAGAAGATCTGGTGTCTCTGCAGCTGGCCTA
CGACGACGACCTGAAGGCCATCTGGTCTAGCTACCCTGATGTGGCCCAGGCCGAGG
ATACAATGGCTGCCGCTGAAGCTGATGCCGTGGCTCTGTCTGAGAGAGTGAAGCAG
GCCAGAATCGAGGCCAGAAGCAAGAAGATCAGCACCGAGCTGACCCAGCAGCTGA
GGGACGCCAAGAAGAGACTGAAGGACGCCAGACAGGCCAGACGGGATGCCATTGC
CGTGGTCAAAGATGATGCCGCCGAGCGGAGAAAGGCCAGATCTGATCAACTGGCCG
CCGACCAGAAAGCCCTGTACGGCCAGTACTGCAGAGATGGCGATCTGTACTGGGCC
AGCTTCAACACCGTGCTGGACCACCACAAGACCGCCGTGAAGAGAATCGCCGCTCA
GAGAGCCTCTGGCAAGCCTGCCACACTGAGACACCACAGATTTGACGGCAGCGGCA
CAATCGCCGTGCAACTCCAAAGACAAGCTGGCGCCCCTCCTCGGACACCTATGGTTC
TTGCTGATGAGGCCGGCAAGTACCGGAACGTGCTGCACATTCCTGGCTGGACCGATC
CTGATGTGTGGGAGCAGATGACACGGTCCCAGTGCAGACAGTCTGGCAGAGTGACA
GTGCGGATGAGATGCGGCAGCACAGATGGACAGCCCCAGTGGATCGATCTGCCTGT
GCAGGTTCACAGATGGCTGCCTGCCGACGCCGATATTACAGGCGCTGAGCTGGTGG
TCACAAGAGTGGCCGGAATCTACAGAGCCAAGCTGTGCGTGACCGCCAGGATCGGA
GATACCGAGCCTGTGACATCTGGCCCTACAGTGGCCCTGCATCTTGGCTGGCGATCT
ACCGAAGAGGGAACAGCCGTGGCCACTTGGAGATCTGATGCCCCTCTGGACATCCC
CTTCGGCCTGAGAACAGTGATGAGAGTGGATGCTGCCGGCACCTCTGGCATCATTGT
GGTGCCTGCCACCATCGAGAGAAGGCTGACCCGGACAGAGAATATCGCCAGCAGCA

GATCTCTGGCCCTGGACGCCCTGAGAGATAAGGTTGTCGGCTGGCTGAGCGACAAC
GACGCCCCTACCTATAGAGATGCTCCACTGGAAGCCGCCACCGTGAAGCAGTGGAA
GTCCCCTCAGAGATTCGCCTCTCTGGCTCACGCCTGGAAGGACAATGGCACCGAGAT
CAGCGATATCCTGTGGGCCTGGTTCAGCCTGGACAGAAAGCAGTGGGCTCAGCAAG
AGAACGGCAGAAGAAAGGCCCTGGGACACAGAGATGACCTGTATCGGCAGATCGC
CGCCGTGATCTCTGATCAGGCTGGACACGTGCTGGTGGACGATACAAGTGTGGCCG
AGCTGAGCGCCAGAGCTATGGAAAGAACCGAGCTGCCTACCGAGGTGCAGCAGAA
GATCGACAGAAGAAGGGACCACGCTGCTCCCGGTGGACTGAGAGCTTCTGTGGTGG
CCGCCATGACAAGAGATGGGGTGCCAGTGACAATCGTGGCCGCAGCCGATTTCACC
CGGACACACAGCAGATGCGGACACGTGAACCCCGCCGACGATAGATACCTGAGCAA
CCCCGTCAGATGCGACGGCTGTGGCGCTATGTACGACCAGGACAGATCCTTCGTGAC
CCTGATGCTGAGAGCCGCCACAGCTCCTAGCAATCCTGGCCCAGCCCCAGCCCCAGC
CAGCGAAGTGGAATTCAGCCATGAATATTGGATGCGTCATGCACTGACCCTGGCAA
AACGTGCCCGTGATGAAGGTGAAGCACCGGTTGGTGCAGTTCTGGTTCTGAATAATC
GTGTTATTGGTGAAGGTTGGAATCGTCGTATTGGTCTGCATGATCCGACCGCACATG
CAGAAATTATGGCACTGCGTCAAGGTGGTCTGGTTATGCAGAATAGCCGTCTGATTG
ATGCAACCCTGTATGTTACCTTTGAACCGTGTGTTATGTGTGCCGGTGCAATGATTA
ATAGCCGCATTGGTCGTGTTGTTTTTGGTGTTCGTAATAGCAAACGTGGTGCAGCAG
GTAGCCTGATGAATGTTCTGAATTATCCGGGTATGAATCACCGTGTTGAAATTACCG
AAGGTATTCTGGCAGATGAATGTGCAGCACTGCTGTGTGATTTTTATCGTATGCCTC
GTCAGGTTTTAACGCACAGAAAAAGGCACAGAGCAGCATTAATAAAAGACCTGCC
GCCACAAAGAAGGCCGGACAGGCCAAAAAGAAAAAGCATCACCATCACCATCAC

Supplementary Figure S7. The complete nucleotide sequence coding for the Gag-MmCas12m-TadDE-6xHis tag fusion protein.

Black = HIV-1 Gag; Blue = the *Mycolicibacterium mucogenicum* Cas12m; Violet = the PAPAPA linker; Green = the TadDE, Orange = nucleoplasmin NLS, Red = 6xHis tag.

F-Target_1	CACGGCAAAACCCCCGTCA
R-Target_1	TTCCTCCACCCTGCATAGC
F-Target_2	AAAACTAGGCTGTCCTGGGC
R-Target_2	TGCCTTTTGGGTGGATTCTC
F-Target_3	CACCCAGACCTGACCCAAAC
R-Target_3	CCCAGTGCTATCTGGGACA
F-Target_4	AACCCCTGTAGGGAAGGGG
R-Target_4	TCGTGGCCGCCTCTACTCC
F-Target_5	TACTGGCCTTATCTCACAGG
R-Target_5	TGTCTGGTGCGTTTCACTG
F-Target_6	AAGGAAGCTGCAGCACCAG
R-Target_6	CCCATTTCCTGGAGCCATCTC
F-Target_7	GACGCAAGGGAGACATCCG
R-Target_7	TTCCTCCACTCCCTCTTCCC
F-(C/A)10-motif	TCTGGTGGAACCCCAAGATGG
R-(C/A)10-motif	AGCCACAGACATGATTGCAAATACAC

Supplementary Figure S8. Primers used in this study to amplify target sites for NGS.

Cas12-dBE:

C1	A2	C3	A4	C5	C6	A7	A8	C9	C10	C12	C13	C16	C17
2.25	4.21	7.13	12.5	14.4	10.1	6.82	3.52	2.55	1.2	0.75	0	0	0
2.74	5.01	7.83	11.23	15.9	10.51	5.8	3.03	2.02	0.95	1.09	0	0	0
2.95	4.72	7.23	10.65	14.7	11.4	4.93	3.25	2.31	1.37	0.85	0	0	0

Cas9-dBE:

C1	A2	C3	A4	C5	C6	A7	A8	C9	C10	C12	C13	C16	C17
0	10.4	14.3	24.2	38.51	34.5	17.2	8.4	2.5	0	0	0	0	0
0	9.36	13.23	23.31	39.8	35.01	18.12	7.87	3.01	0	0	0	0	0
0	10.9	13.7	24.5	38.2	33.1	18.61	7.49	2.27	0	0	0	0	0

dBE-Cas12:

C1	A2	C3	A4	C5	C6	A7	A8	C9	C10	C12	C13	C16	C17
3.05	4.54	9.12	11.5	15.2	10.8	7.54	4.41	2.9	1.35	1.57	0.9	0.51	0.06
2.87	4.02	8.21	12.12	14.02	11.2	7.1	4.5	3.12	1.02	1.34	1.03	0.25	0.1
3.56	4.3	8.02	11.32	14.5	10.01	8.01	4.21	2.71	1.03	1.48	0.85	0,67	0.09

dBE-Cas9:

C1	A2	C3	A4	C5	C6	A7	A8	C9	C10	C12	C13	C16	C17
7.02	24.8	33.23	35.9	39.2	34.2	28.2	17.21	7.21	4.3	0	0	0	0
6.5	25.7	34.9	36.2	40.4	34.82	29.5	16.72	8.32	3.41	0	0	0	0
6.78	26.02	34.3	37.5	39.21	33.5	28.43	18.02	9.03	3.71	0	0	0	0

Supplementary Figure S9. Comparison of N- and C-terminal deaminase fusions to MmCas12m and dSpCas9. Source data.

XTEN:

C1	A2	C3	A4	C5	C6	A7	A8	C9	C10	C12
2.25	4.21	7.13	12.5	15.4	11.1	6.82	3.52	2.55	1.2	0.75
2.74	5.01	7.83	11.23	16.52	10.51	5.8	3.03	2.02	0.95	1.09
2.95	4.72	7.23	10.65	15.17	12.4	4.93	3.25	2.31	1.37	0.85

(SGGS)3:

C1	A2	C3	A4	C5	C6	A7	A8	C9	C10	C12
1.52	4.11	7.13	11.56	16.54	10.53	6.71	4.31	2.81	0.85	0.75
1.44	4.57	7.61	10.92	14.92	10.26	7.38	4.63	2.56	0.65	0.91
1.25	3.71	7.54	11.78	15.71	12.32	6.43	3.92	2.43	0.91	0.64

(SGGS)2:

C1	A2	C3	A4	C5	C6	A7	A8	C9	C10	C12
0	1.41	5.83	11.52	14.72	10.22	6.12	4.32	1.31	0	0
0	2.64	6.51	10.55	16.31	9.45	5.64	3.73	1.54	0	0
0	1.33	5.15	11.03	15.01	11.89	6.51	3.56	1.12	0	0

SGGS:

C1	A2	C3	A4	C5	C6	A7	A8	C9	C10	C12
0	0	2.53	10.05	15.1	8.81	4.14	1.21	0	0	0
0	0	1.71	11.09	14.05	9.12	3.52	1.56	0	0	0
0	0	2.15	11.52	13.62	7.74	3.37	1.43	0	0	0

SG:

C1	A2	C3	A4	C5	C6	A7	A8	C9	C10	C12
0	0	0	1.43	1.51	1.15	0	0	0	0	0
0	0	0	1.21	0.76	0.62	0.21	0	0	0	0
0	0	0.24	1.09	0.52	0.97	0.12	0	0	0	0

P(AP)8:

C1	A2	C3	A4	C5	C6	A7	A8	C9	C10	C12
0	0.32	2.11	4.19	15.21	10.53	0	0	0	0	0
0	0.47	1.7	3.73	16.78	9.45	0.21	0	0	0	0
0	0.54	1.92	4.61	14.41	8.51	0.09	0	0	0	0

P(AP)4:

C1	A2	C3	A4	C5	C6	A7	A8	C9	C10	C12
0	0	0.35	4.91	14.52	7.13	0	0	0	0	0
0	0	0.51	4.13	13.09	8.82	0.1	0	0	0	0
0	0	1.02	3.85	16.73	6.61	0	0	0	0	0

PAPAPA:

C1	A2	C3	A4	C5	C6	A7	A8	C9	C10	C12
0	0	0	0.09	14.05	0.1	0	0	0	0	0
0	0	0	0.31	14.41	0.28	0	0	0	0	0
0	0	0	0.2	13.99	0.37	0	0	0	0	0

PAPA:

C1	A2	C3	A4	C5	C6	A7	A8	C9	C10	C12
0	0	0	0	4.52	0.11	0	0	0	0	0
0	0	0	0	5.14	0.24	0	0	0	0	0
0	0	0	0	4.91	0.16	0	0	0	0	0

No linker:

C1	A2	C3	A4	C5	C6	A7	A8	C9	C10	C12
0	0.11	0	0	0.1	0.13	0	0	0	0	0
0	0.23	0	0	0.22	0.2	0	0	0	0	0
0	0	0	0	0.13	0.11	0	0	0	0	0

Supplementary Figure S10. Comparison of different linkers. Editing efficiency and specificity of the Cas12-dBE base editors tested by the target $(C/A)_{10}$ motif. Source data.

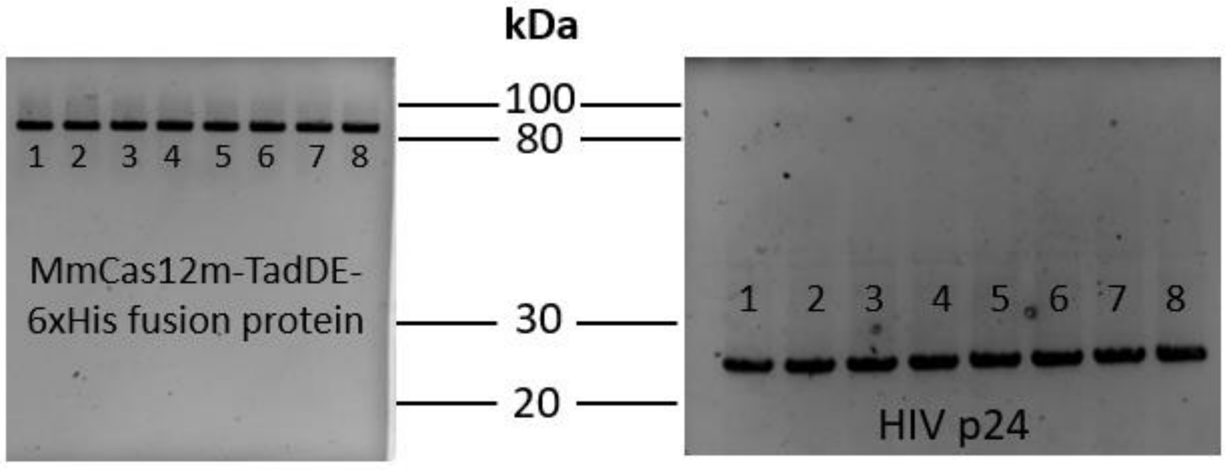

Supplementary Figure S11. Western blot confirming the content of MmCas12m-TadDE editing complexes (about 86 kDa) in VLP for each of the eight AAVS1 targets. HIV p24 (24 kDa) serves as a load control.

Target 1 AAVS1:

A1	A2	C3	C4	C5	A6	C8	C9	A10	C14	C15
0	0	0	0.16	7.82	0.13	0	0	0	0	0
0	0	0	0	9.41	0.12	0	0	0	0	0
0	0	0	0	8.62	0.2	0	0	0	0	0

Target 2 AAVS1:

C5	C6	A7	C8	A11	A12	C13	C14	A17	A19	A20
10.15	0	0	0	0	0	0	0	0	0	0
10.95	0.12	0	0	0	0	0	0	0	0	0
9.51	0	0	0	0	0	0	0	0	0	0

Target 3 AAVS1:

C1	C2	A3	A4	C6	A7	C10	A11	C12	C13	C14
0	0	0	0.12	0	0	0	0	0	0	0
0	0	0	0	0	0	0	0	0	0	0
0	0	0	0	0	0	0	0	0	0	0

Target 4 AAVS1:

A4	A5	C7	A11	C12	C13	C14	C17	C19
0	8.51	0	0	0	0	0	0	0
0	7.4	0	0	0	0	0	0	0
0	6.73	0	0	0	0	0	0	0

Target 5 AAVS1:

C3	C4	A5	A6	A7	C8	C11	C16	C17	C19
0	0.77	8.73	0.32	0	0	0	0	0	0
0	0.75	8.11	0.53	0	0	0	0	0	0
0	0.35	7.56	0.21	0	0	0	0	0	0

Target 6 AAVS1:

A5	A7	A12	C14	C16	A17
6.73	0	0	0	0	0
8.06	0	0	0	0	0
9.52	0	0	0	0	0

Target 7 AAVS1:

A5	A9	A12	A14	A16	C19	C20
8.96	0	0	0	0	0	0
9.92	0	0	0	0	0	0
10.51	0	0	0	0	0	0

(C/A)10 motif AAVS1:

C1	A2	C3	A4	C5	C6	A7	A8	C9	C10	C12
0	0	0	0	8.4	0.1	0	0	0	0	0
0	0	0	0.23	9.52	0.51	0	0	0	0	0
0	0	0	0.15	7.97	0	0	0	0	0	0

Supplementary Figure S12. The efficiency of non-stoichiometrically improved VLP-MmCas12m-mediated editing of target sites in the AAVS1 locus. Source data.

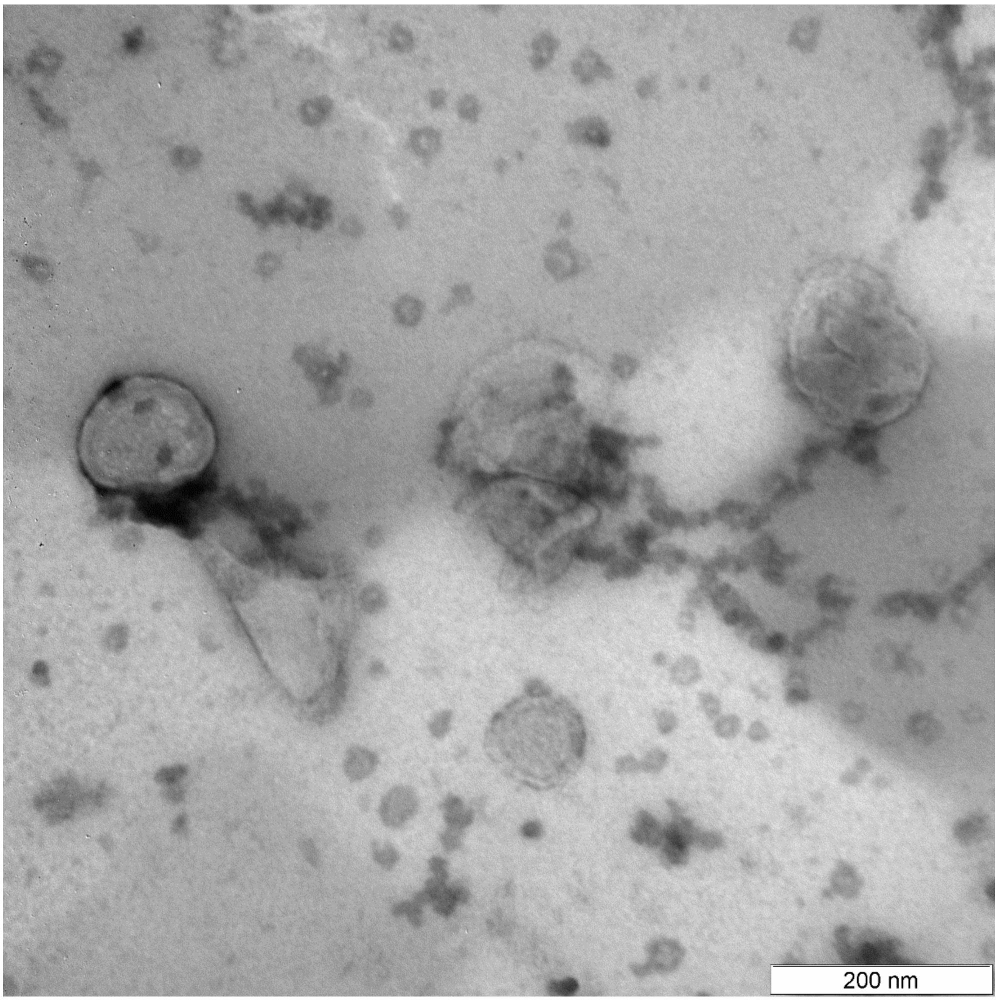

Supplementary Figure S13. The electron microscopic image of stoichiometrically improved Virus-like particles.

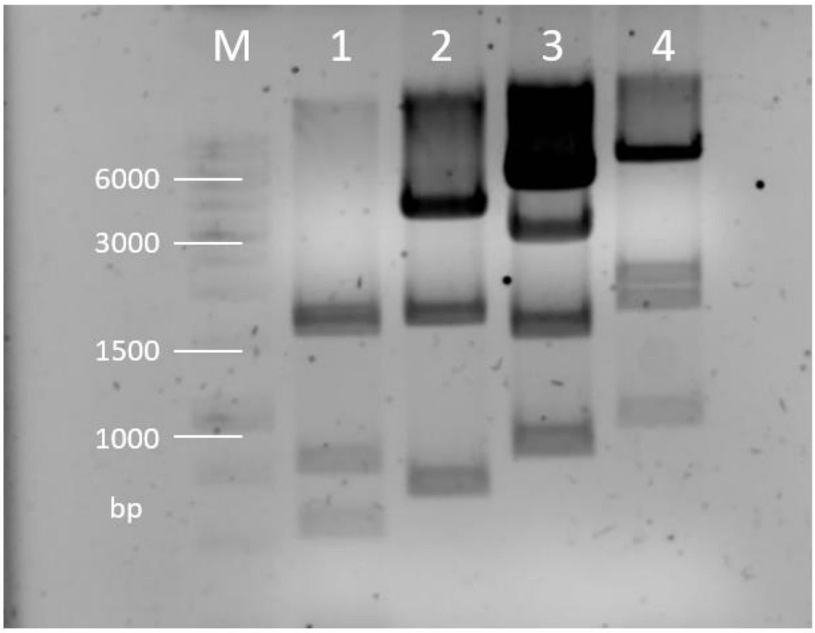

Supplementary Figure S14. Restriction digest of plasmids used for VLP production.

M – 1 kb DNA marker, 1 – psgRNA-MmCas12m (digested by PvuI, NdeI), 2 – pCMV-VSV-G (digested by StuI, EcoRI), 3 – pLentiPack (digested by SpeI, XbaI), 4 – pGag-MmCas12m-TadDE (digested by XhoI, StuI).

Site name	Spacer (mismatches are highlighted in red)	Chromosome	Position	Primers for amplification
OT1-sgRNA1	AtCCCAGCCAatTCCTTCCA	15	27216010	AATAATCTATACTCGTGTCTTTTTC<br>GAATTTGGTCTGCAATGCCCACAAA
OT2-sgRNA1	AAaCCAGtCAGGTCCTTCaA	1	30919172	ATCCTCAAATCCATCCCTCCGAGGAG<br>ATCACGAGACCCTGTTACAGGAC
OT3-sgRNA1	AcCCCAGCCAGGTaCTTCtA	1	179848617	CAAATTTGAAACTCTTAGTGAAGG<br>CTTTAATATCCCTGGACCTTTTTT
OT4-sgRNA1	AgCCCAGCCAGGcCCTcCCA	22	44281381	CCTGAGGGGAGAGAAGGCTCAG<br>TCAGGACAACCCCTGAGGGCCC
OT1-sgRNA2	TaGCCACGTgACCTGAGAg	8	50765307	GGGGAAAAATGCCCAAGCTTAT<br>TGGGGTTATCAAGAGTGAGAAACT
OT2- sgRNA2	TGGCgACaTAACCTGAGAg	8	88005516	ACAAATGAGGAGGAGTCCTA<br>TATTTGTGCTTTTCCCAAGTTTCC
OT3- sgRNA2	TGGCCACGTgACCTaAGAc	5	73302948	TGTTTTACATTCATTTATG<br>AACCTACGGATAAGGCCCCAA
OT1-sgRNA7	GGTGAaGGAGGAGAGActCC	15	39180165	CTCCAAGCTGATCATCTATTCCAT<br>CTTAATCTTTTTGGCTTGATGTAA
OT2-sgRNA7	aGTGAGGcAGaAGAGATGCC	5	106253751	TAAATGTTGTGGTAATTCTAAAAG<br>ACCATATAAATATTTATTCAAAAA
OT3-sgRNA7	GGTGtGGGAGcAGAGtTGCC	20	46926824	ATACCTAGAAATAATGTTTCATCTG<br>TCCTATGTGTTGGTAAACTC
OT1-sgRNA(C/A)10	CACACaAACCTgCTTCCCCt	8	24146098	GGCTTTGGGTAAAATGGATAC<br>GGAGGTGGATCCTTTATGAAT
OT2-sgRNA(C/A)10	CtCcCCAACCTCCTaCCCCC	8	24942073	CCAAAACTGCATTTCCAACCAGC<br>GAGAGCCACGCCCCAGCCGCTC
OT3-sgRNA(C/A)10	CAacCCAACCTCCaTCCCCC	8	135397649	GGATGCACTCCTAAGTTC<br>ATTCATTGCATGAATTCATG
OT4-sgRNA(C/A)10	CACACCAACgcCCgTCCCCC	5	3592187	GATGTAAATATGCCCCAGGA<br>CTTGGGCCCCAGTGCTCCGG

Supplementary Figure S15. The list of potential off-target sites during sgRNA1, 2, 7, and sgRNA(C/A)$_{10}$-mediated editing.
